# Identification of CD203c as a New Basophil-Specific Flow-Marker in Ph^+^ Chronic Myeloid Leukemia

**DOI:** 10.3390/cells12010003

**Published:** 2022-12-20

**Authors:** Irina Sadovnik, Daniel Ivanov, Dubravka Smiljkovic, Gabriele Stefanzl, Lina Degenfeld-Schonburg, Susanne Herndlhofer, Gregor Eisenwort, Alexander W. Hauswirth, Thamer Sliwa, Felix Keil, Wolfgang R. Sperr, Peter Valent

**Affiliations:** 1 Department of Internal Medicine I, Division of Hematology and Hemostaseology, Medical University of Vienna, 1090 Vienna, Austria; 2 Ludwig Boltzmann Institute for Hematology and Oncology, Medical University of Vienna, 1090 Vienna, Austria; 3 Third Medical Department for Hematology and Oncology, Hanusch Hospital Vienna, 1140 Vienna, Austria

**Keywords:** CML, BCR::ABL1, basophils, E-NPP3, CD203c, flow cytometry

## Abstract

Basophilia is a crucial prognostic variable in Ph-chromosome-positive chronic myeloid leukemia (CML). The ectoenzyme CD203c is an activation-linked surface antigen that is expressed specifically on basophil-committed progenitor cells and mature basophils. We examined the expression of CD203c on progenitors and/or basophils in 21 healthy donors and 44 patients with CML. As expected, the numbers of CD203c^+^ blood leukocytes were significantly higher in CML patients compared to controls (percentage of CD203c^+^ cells among viable cells in CML at diagnosis: 4.19 ± 3.68% vs. controls: 0.53 ± 0.23%, *p* < 0.05). Moreover, CML basophils expressed higher levels of CD203c compared to normal basophils (median staining-index in CML at diagnosis: 29.41 ± 19.14 versus controls: 20.44 ± 13.45). We also found that the numbers and percentage of circulating CD203c^+^ cells at diagnosis correlate with the disease-related risk-profile. Incubation of CML basophils with an anti-IgE-antibody resulted in further upregulation of CD203c. After successful treatment with imatinib and/or other BCR::ABL1 inhibitors leading to major or complete molecular responses, the numbers of CD203c^+^ basophils decreased substantially in our CML patients compared to pre-treatment values. Together, CD203c is overexpressed on CML basophils, is further upregulated by IgE receptor cross-linking, and may serve as a biomarker to quantify basophilia in patients with CML at diagnosis and during therapy.

## 1. Introduction

Chronic myeloid leukemia (CML) is a myeloid malignancy characterized by an expansion and accumulation of myelopoietic cells and their progenitors in the bone marrow (BM) and peripheral blood (PB). Clonal cells in CML exhibit the disease-related (pathognomonic) reciprocal translocation t(9;22) [1]. The resulting protein–product, the oncoprotein BCR::ABL1, serves as a critical driver of oncogenesis, disease evolution, and progression in CML [2,3]. The clinical course in CML is separable into a chronic phase (CP), accelerated phase (AP), and a blast phase (BP) [3,4]. During CP, the clinical course of CML is usually indolent. Most patients present with a left shift in their differential counts as well as basophilia. In a majority of these patients, the disease responds well to BCR::ABL1-targeting tyrosine kinase inhibitors (TKI), including imatinib, unless *BCR::ABL1* mutations or other genetic lesions causing resistance are present [5,6,7]. By contrast, in AP, the disease is more difficult to control with standard therapy and may transform to BP [2,3,4,8,9,10].

One typical feature of disease-acceleration in CML is a marked or even excessive increase in basophils [11,12]. It has also been reported that basophilia at diagnosis is one of the most powerful (predictive) prognostic markers in CML [13,14,15]. However, in many cases, it is difficult to define the exact number of basophils as these cells are often immature and hypo-granulated in these patients [16,17]. Basophil precursor cells may even exhibit a blast-like morphology, especially when the disease is progressing to AP. As a result, these cells may escape microscopic detection, and it is difficult to exactly quantify the basophil compartment in these cases. Therefore, several attempts have been made to improve basophil counting in CML by application of more sensitive stains or novel basophil-related markers [16,17,18,19].

The cell surface enzyme ectonucleotide pyrophosphatase/phosphodiesterase-3 (E-NPP3), also known as CD203c, has been identified as a cell surface antigen that is specifically expressed on normal blood basophils and their progenitors and less abundantly on tissue mast cells [20,21,22]. In response to cross-linking of the IgE receptor, the cell surface levels of CD203c on basophils increase [23,24,25]. Therefore, CD203c is widely used as an activation-linked marker to quantify basophil responses to allergens. In addition, CD203c has been applied to enumerate basophils and their precursor cells and to purify these cells [20,21,26,27]. However, so far, CD203c has not been analyzed in detail in the context of CML.

The aims of our project were to examine whether CD203c is a reliable biomarker of CML basophils and their progenitors, to explore the regulation of expression of CD203c in CML basophils, and to define the prognostic impact of CD203c.

## 2. Materials and Methods

### 2.1. CML Patients and Controls

A total number of 44 patients with CML (at diagnosis, *n* = 39; diagnosis and follow-up, *n* = 18; samples collected only during follow-up: *n* = 5) were analyzed. At diagnosis, patients were classified as CP (*n* = 36) and AP (*n* = 3). The patients’ characteristics are shown in Table S1. Mononuclear cells (MNC) were isolated from heparinized PB or BM samples using Ficoll. *BCR::ABL1* mRNA was quantified in leukemic cells at diagnosis and in the follow-up. *BCR::ABL1* mRNA was determined according to the international scale and expressed as percentage of *ABL1* mRNA. The numbers of basophils and the numbers of CD203c^+^ cells in the PB (percentage and absolute numbers/µL blood) were examined at diagnosis (*n* = 29) and/or in the follow-up (*n* = 23). In a subset of patients (*n* = 22), we also determined serum tryptase levels at diagnosis by a commercial fluoroenzyme-immunoassay essentially as described [19,28,29]. We also measured the percentage of CD203c^+^ blood leukocytes in 21 healthy controls. In addition, control BM cells, including normal/reactive BM or BM of patients with lymphoid neoplasms and other myeloid neoplasms, including myeloproliferative neoplasms (MPN), myelodysplastic syndrome (MDS), chronic myelomonocytic leukemia (CMML), and acute myeloid leukemia (AML) were analyzed (*n* = 11). For in vitro experiments, CML MNC and the *BCR::ABL1*+ human basophil cell line KU812 were employed. Details are described in the Supplementary Materials. All patients provided written informed consent before PB or BM were obtained. All studies were approved by the ethics committee of the Medical University of Vienna.

### 2.2. Monoclonal Antibodies (mAb) and Other Reagents

Reagents used in this study are described in the Supplementary Materials. The PE-labeled mAb 97A6 (CD203c) and the FITC-labeled mAb CLBGran/12 (CD63) were purchased from Beckman Coulter (Brea, CA, USA) and the anti-IgE mAb E124.2.8 (Dε2) was purchased from Sigma-Aldrich (Saint Louis, MO, USA). A characterization of mAb used in this study is provided in Table S2.

### 2.3. Multi-Color Flow Cytometry

Multi-color flow cytometry was performed on BM or PB cells (whole blood or Ficoll-isolated MNC) and KU812 cells using mAb essentially as described [23,24,25,26]. Basophils were identified as CD123^+^/CD45^+^/CD203c^+^ cells and gated as shown in Figure S1. Expression of CD203c on basophils was determined by measuring the median fluorescence intensity (MFI) produced by the CD203c mAb and an isotype-matched control mAb. The staining index (SI) was calculated using the formula: MFI CD203c divided by MFI of isotype-control mAb. In four patients with CML, we examined BM cells by multi-color flow cytometry and determined CD203c expression on CD34^+^/CD45^+^ stem- and progenitor cells and CD123^+^/FcεRIα^+^ basophils. In a separate set of experiments, we compared the expression-levels of CD203c on CD203c^+^/CD123^+^/FcεRIα^low^ and CD203c^+^/CD123^+^/FcεRIα^high^ basophil subsets. Flow cytometric data were acquired on a FACSCalibur (BD Biosciences, San José, CA, USA), FACSCantoII (BD Biosciences), or a CytoFLEX S (Beckman Coulter).

### 2.4. Purification of CML Basophils and Quantitative PCR (qPCR)

In three patients with CML (CP, *n* = 2, AP, *n* = 1), basophils were purified to homogeneity from PB MNC by cell sorting essentially as reported [20,26]. In a first step, basophils were enriched by magnetic cell sorting using the basophil isolation kit II (Miltenyi Biotec, Bergisch, Gladbach, Germany). Then, basophils were purified by cell sorting using PE-labeled CD203c mAb 97A6 on a FACSAria Fusion (BD Biosciences) [26]. The resulting purity of basophils was >95%. qPCR was performed on purified basophils and KU812 cells as described in the Supplementary Materials. *ABL1* was employed as a reference gene.

### 2.5. Incubation of Basophils with Anti-IgE Antibody or with Targeted Drugs

Blood MNC obtained from six patients with CML (CP, *n* = 4, AP, *n* = 2) and seven healthy donors were incubated in control medium or medium supplemented with anti-IgE antibody E124.2.8 (0.01–10 µg/mL) together with fluorochrome-labeled mAb against CD203c, CD63, CD45, and CD123 at 37 °C for 15 min. Then, cells were washed, and the expression of CD203c and CD63 on basophils was analyzed by flow cytometry. In a separate set of experiments, dextran enriched blood basophils from five patients with CML (CP, *n* = 4, AP, *n* = 1) were incubated in control medium or medium containing increasing concentrations of interleukin-3 (IL-3) (0.001–100 ng/mL). Thereafter, cells were incubated with a fluorochrome-labeled mAb against CD203c (37 °C, 15 min) and analyzed using flow cytometry. To study the impact of BCR::ABL1 on expression of CD203c and CD63, MNC obtained from five CML patients (CP, *n* = 3, AP, *n* = 2) were incubated in control medium or medium containing IL-3 (1 ng/mL) and various concentrations of the BCR::ABL1-targeting drugs imatinib, nilotinib, or bosutinib (0.1, 0.5 or 1 µM) at 37 °C for 48 h. Thereafter, expression of CD203c on basophils was analyzed by flow cytometry. KU812 cells were grown in RPMI 1640 medium supplemented with 10% FCS and antibiotics at 37 °C. To study the potential involvement of signal transduction molecules in expression of CD63 and CD203c, KU812 cells were exposed to various targeted drugs, including nilotinib, imatinib, bosutinib, ibrutinib, masitinib, midostaurin, avapritinib, BEZ235, asciminib, copanlisib, fedratinib, or rapamycin (0.005–5 µM) at 37 °C for 48 h. After incubation, expression of CD63 and CD203c was analyzed by flow cytometry. Technical details are described in the Supplementary Materials.

### 2.6. Statistical Analysis

To calculate correlations and the levels of significance between two non-parametric data, the Spearman’s rank correlation coefficient was used. The Mann–Whitney U test was applied when two independent variables were compared (e.g., comparing expression levels of CD203c in CML versus healthy PB donors). When comparing CD203c expression data between dependent groups (e.g., data analyzed from the same patients at diagnosis and follow-up) the paired *t*-test was applied. When comparing more than two variables either the Kruskal–Wallis test or ANOVA with Dunnett’s post hoc adjustment was applied. Differences were considered to be significant when *p* < 0.05.

## 3. Results

### 3.1. CD203c Is a Novel Biomarker of Blood Basophils in CML

**Figure 1:**
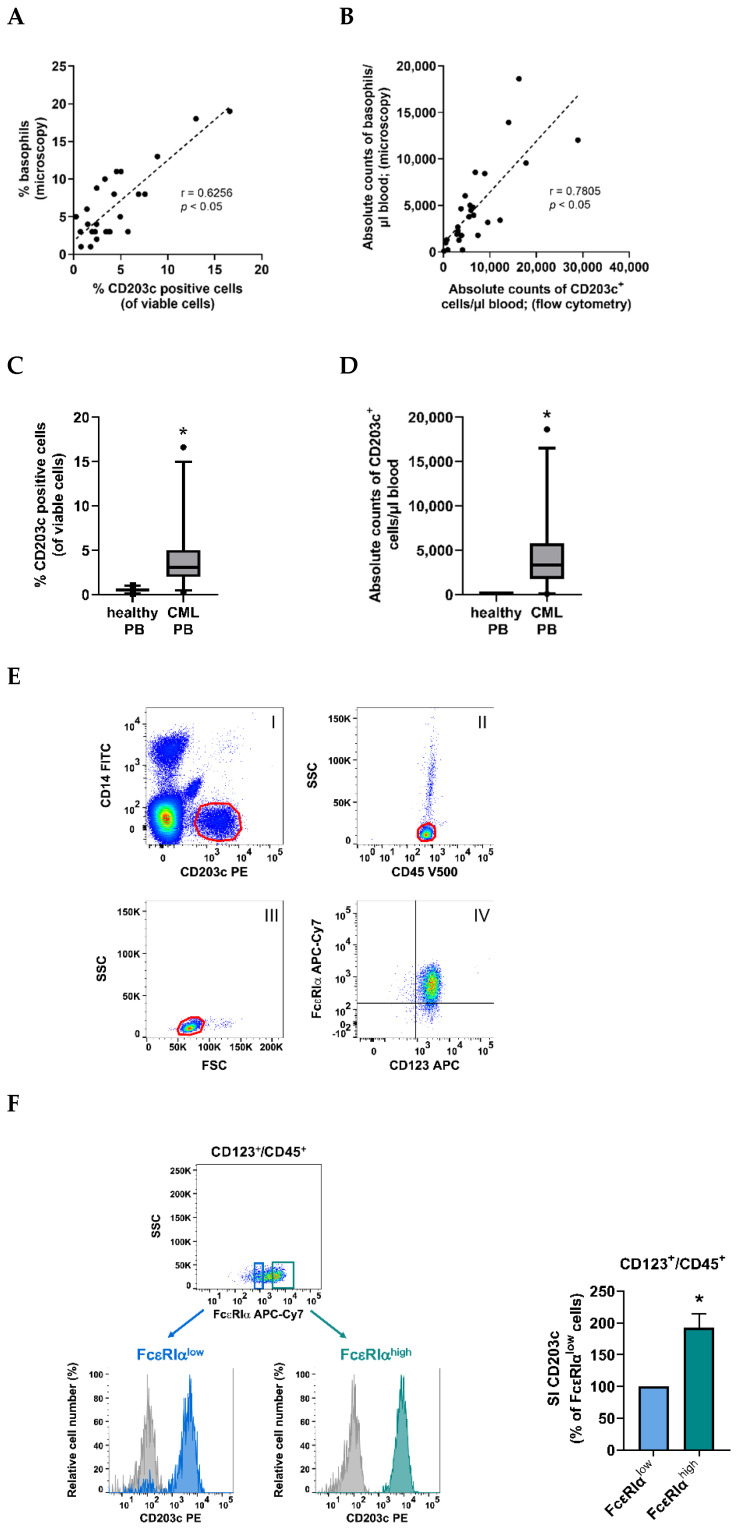
CD203c as a novel specific biomarker for basophils in CML. (**A**) Correlation between percentage (%) of microscopic basophils and % of CD203c positive cells by flow cytometry in patients with CML (*n* = 26). (**B**) Correlation of absolute basophil numbers (microscopic basophils/µL blood determined from differential counts and white blood counts) and absolute numbers of CD203c^+^ cells/µL blood in 26 CML patients. (**A**,**B**): *p*, *p*-value; r, Spearman’s rank correlation coefficient. (**C**) Flow cytometric analysis of the percentage (%) of CD203c^+^ cells in peripheral blood (PB) samples from CML patients (*n* = 28) compared with normal PB samples (*n* = 21). The figure shows box and whisker plots (horizontal lines: median values; boxes: 25/75 percentiles; whiskers: 5/95 percentiles; dot: outlier). (**D**) Absolute numbers of CD203c^+^ cells/µL blood in PB samples from 28 CML patients and 11 healthy donors. The figure shows box and whisker plots (horizontal lines: median values; boxes: 25/75 percentiles; whiskers: 5/95 percentiles; dot: outlier). (**C**,**D**): asterisk (*): *p* < 0.05 by Mann–Whitney U test. (**E**) Demonstration of co-expression of FcεRIα on CD123^+^ and CD203c^+^ cells in CML cells (patient #18A). In a first step, basophils were gated by expression of CD203c and exclusion of CD14^+^ cells (I). Then, cells were gated as CD45^+^/SSC^low^ cells (II). Thereafter, viable cells were selected by their light-scatter properties (III). The final gate was set to identify FcεRIα on CD123^+^ cells co-expressing CD203c (IV). (**F**) The dot plot in the upper left panel shows the gating for the FcεRIα^low^ (blue gate) and FcεRIα^high^ (green gate) fractions within the CD123^+^/CD45^+^/CD14^–^ cells in a CML patient (#37A). The lower panels show CD203c expression on FcεRIα^low^ (blue histogram, left panel) and FcεRIα^high^ (green histogram, right panel) cells. Gray histograms represent the isotype control. The right panel shows the staining index (SI) values of CD203c in the FcεRIα^high^ fraction of CD123^+^/CD45^+^/CD14^–^ cells (green bar) relative to CD203c SI values (100%) of the FcεRIα^low^ fraction (blue bar) in four CML patients (#28A, #37A, #38A, #39A). SI values were calculated as median fluorescence intensity (MFI) of CD203c divided by MFI of the isotype control. Results are expressed as mean ± S.D. from four donors. Asterisk (*): *p* < 0.05 by paired *t*-test.

The percentage of CD203c^+^ cells in the PB of our patients with CML was found to correlate with the percentage of basophils recorded by microscopy (Figure 1A). A correlation was also found when comparing absolute numbers of basophils detected by microscopy and absolute numbers of CD203c^+^ cells (Figure 1B). The percentage and absolute numbers of CD203c^+^ cells (basophils) were found to be higher in patients with CML (4.19 ± 3.68%; 4654.94 ± 4445.49 basophils/µL blood) compared to healthy controls (0.53 ± 0.23%; 35.14 ± 12.48 basophils/µL blood) (*p* < 0.05) (Figure 1C,D). The identity of basophils was confirmed by demonstrating co-expression of CD123 and FcεRIα on CD203c^+^ cells (Figure 1E). Other cells in the CML samples examined, including CD14^+^ monocytes, CD16^+^ neutrophils, CD3^+^ T cells, CD19^+^ B cells, and CD45^+^/SSC^high^/FSC^high^ eosinophils, did not express CD203c (Table 1). We also examined patients with other myeloid neoplasms, including myeloproliferative neoplasms, chronic myelomonocytic leukemia, and acute myeloid leukemia. Again, as in CML, only basophils were found to stain positive for CD203c, whereas the other cell types examined stained negative for CD203c (data not shown). In the BM of our patients with CML, CD203c was detected on a subset of CD34^+^/CD45^+^ progenitor cells, basophils, and CD117^+^/CD34^−^ mast cells (Table 1, Figure S2). The percentage counts of CD203c^+^ cells among CD34^+^/CD45^+^ CML cells ranged between 3.4% and 21.7% and were thus higher than the percentage counts of CD203c^+^ stem- and progenitor cells in normal BM samples (range: 0.7%–1.5%) (Figure S2). As expected, expression levels of CD203c were higher on CD123^+^/FcεRIα^high^ (more mature) BM basophils than on CD123^+^/FcεRIα^low^ (presumably more immature) BM basophils (Figure 1F) in the CML samples tested. Other cells in control BM samples or in CML BM samples did not stain positive for CD203c (Table 1). To confirm expression of CD203c in basophils at the mRNA level, we performed qPCR experiments on purified (sorted) CML basophils. These cells expressed *CD203c* mRNA and *CD63* mRNA in all donors tested (Table S3). KU812 served as control and also displayed transcripts for *CD203c* and *CD63* (Table S3).

### 3.2. CML Basophils Express Higher Baseline Levels of CD203c Than Normal Basophils

**Figure 2:**
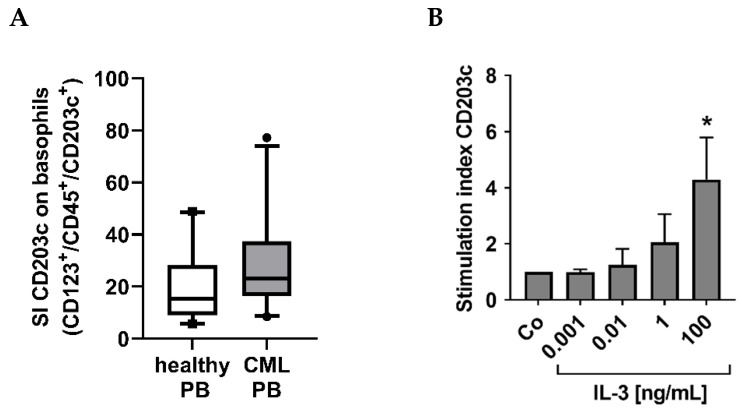
Intensity of CD203c expression on normal and CML basophils. (**A**) Flow cytometric analyses of the intensity of CD203c expression on blood basophils (CD123^+^/CD45^+^/CD203c^+^/CD14^−^) from healthy donors (white box, *n* = 20) and CML patients (gray box, *n* = 28). The figure shows box and whisker plots (horizontal lines: median values; boxes: 25/75 percentiles; whiskers: 5/95 percentiles; dots: outliers) and represents staining index (SI) values of CD203c on basophils determined as median fluorescence intensity (MFI) of CD203c on basophils divided by MFI of the corresponding isotype control. (**B**) CML cells were cultured in control medium (Co) or medium containing increasing concentrations of IL-3 (0.001–100 ng/mL) at 37 °C for 24 h and analyzed for CD203c expression on basophils by flow cytometry. The figures show the stimulation index calculated as mean fluorescence intensities of CD203c after IL-3 exposure divided by the mean fluorescence intensities of CD203c on basophils incubated in control medium. Results represent the mean ± S.D. from five CML patients (#6A, #13A, #30A, #42A, #43A). Asterisk (*): *p* < 0.05 by one way ANOVA with Dunnett’s post hoc test.

As assessed by flow cytometry, CML basophils (*n* = 28 patients) expressed slightly higher levels of CD203c (SI levels) compared to blood basophils in healthy individuals (*n* = 20) (basophil CD203c: SI in CML: 29.41 ± 19.14 vs. SI in controls: 20.44 ± 13.45) (Figure 2A). IL-3 was found to upregulate expression of CD203c on CML basophils (Figure 2B) confirming previous data obtained with normal basophils and basophils of allergic donors [30,31].

### 3.3. The Numbers of CD203c^+^ Cells and the Levels of CD203c on Basophils Decrease after Successful Treatment with Imatinib

**Figure 3:**
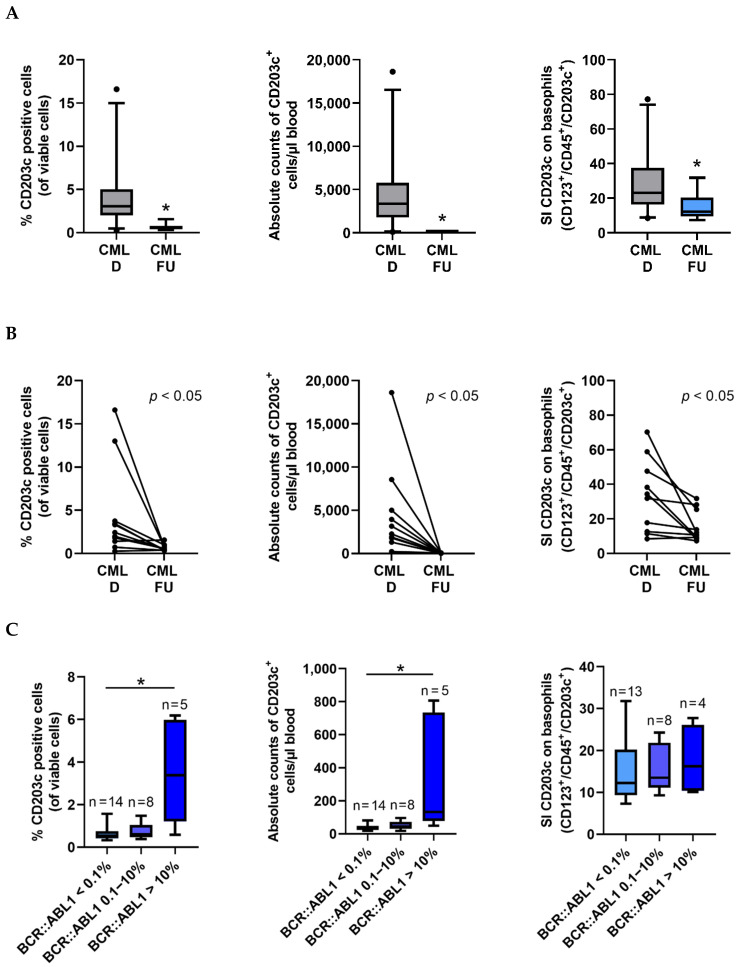

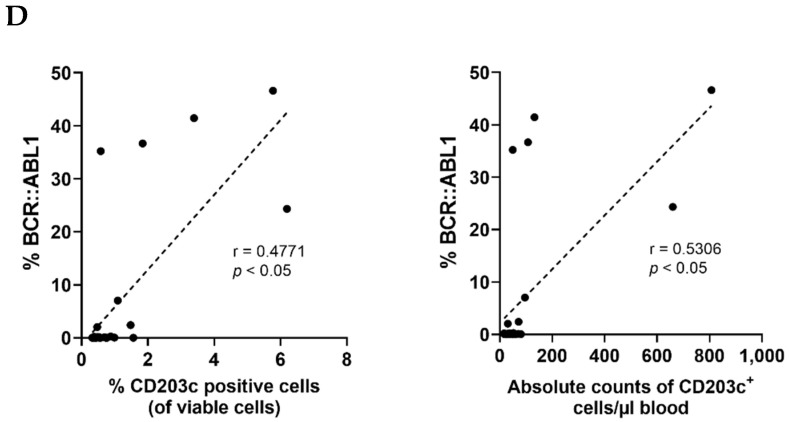
Expression of CD203c and the intensity of CD203c expression on basophils in CML during follow-up. (**A**) Flow cytometric analyses of CD203c expression in peripheral blood (PB) samples from CML patients at diagnosis (CML D; gray box) and follow-up (FU) samples of patients receiving tyrosine kinase inhibitor (TKI) therapy (CML FU; blue box). FU samples were analyzed in patients who received at least 1 month TKI and reached a *BCR::ABL1* value < 0.1%. The left panel shows the percentages (%) of CD203c^+^ cells and the middle panel the absolute numbers of CD203c^+^ cells/µL blood in 28 CML samples at diagnosis and 14 FU samples. The right panel shows the staining index (SI) of CD203c on CD123^+^/CD45^+^/CD203c^+^/CD14^−^ CML cells (basophils) in 28 CML samples at diagnosis and 13 FU samples. The graphs show box and whisker plots (horizontal lines: median values; boxes: 25/75 percentiles; whiskers: 5/95 percentiles; dots: outliers). SI = median fluorescence intensity (MFI) of CD203c on basophils divided by MFI of the isotype control. Asterisk (*): *p* < 0.05 by Mann–Whitney U test. (**B**) The line plots show the flow cytometric analyses of the percent (%) of CD203c^+^ cells (left panel; *n* = 11), the absolute count of CD203c^+^ cells (middle panel; *n* = 11) and the SI values of CD203c on basophils (right panel; *n* = 10) in the same patients at diagnosis and during FU (at least one month TKI and *BCR::ABL1* <0.1%). *p* < 0.05 calculated by paired *t*-test. (**C**) Percentage counts (%) of CD203c^+^ cells (left panel), absolute counts of CD203c^+^ cells (middle panel) and SI of CD203c on basophils (right panel) in CML patients who achieved <0.1% *BCR::ABL1* (light blue boxes), 0.1–10% *BCR::ABL1* (blue boxes), or >10% *BCR::ABL1* (dark blue boxes). The figure shows box and whisker plots (horizontal lines: median values; boxes: 25/75 percentiles; whiskers: 5/95 percentiles). Asterisk (*): *p* < 0.05 by Kruskal–Wallis test. N, numbers of patients analyzed in each group. (**D**) The left panel shows the correlation between % of *BCR::ABL1* and % of CD203c^+^ cells by flow cytometry. The right panel shows the correlation between the % of *BCR::ABL1* and the absolute counts of CD203c^+^ cells. Both graphs show data from all CML patients during follow-up under TKI therapy. Abbreviations: *p*, *p*-value; r, Spearman’s rank correlation coefficient.

Follow-up samples were obtained from 23 patients treated with imatinib, dasatinib, nilotinib, bosutinib, or ponatinib (at least 1 month on TKI therapy). We compared the numbers (percentages and absolute numbers) of CD203c^+^ cells and the amount of CD203c (SI) expressed on basophils at the time of diagnosis and the time of complete response (*n* = 14, at least MR3 = less than 0.1% *BCR::ABL1*). In these studies, we found that the numbers of basophils and the levels of CD203c on basophils significantly decrease during successful treatment with imatinib and/or other TKI (Figure 3A,B). We also found significant differences in the percentages and absolute numbers of CD203c^+^ cells when comparing patients who achieved a deep molecular response (MR3), a substantial decrease in *BCR::ABL1* (0.1–10%) or no substantial decrease in *BCR::ABL1* (>10%) (Figure 3C). By contrast, no significant differences were found when comparing expression levels of CD203c (SI) on basophils in these three groups of patients (Figure 3C, right panel). Finally, we were able to show that the numbers and percentage of CD203c^+^ cells correlate with *BCR::ABL1* mRNA levels when recorded during therapy with imatinib and/or other TKI (Figure 3D).

### 3.4. CML Basophils Express CD203c Independent of BCR::ABL1 Kinase Activity

**Figure 4:**
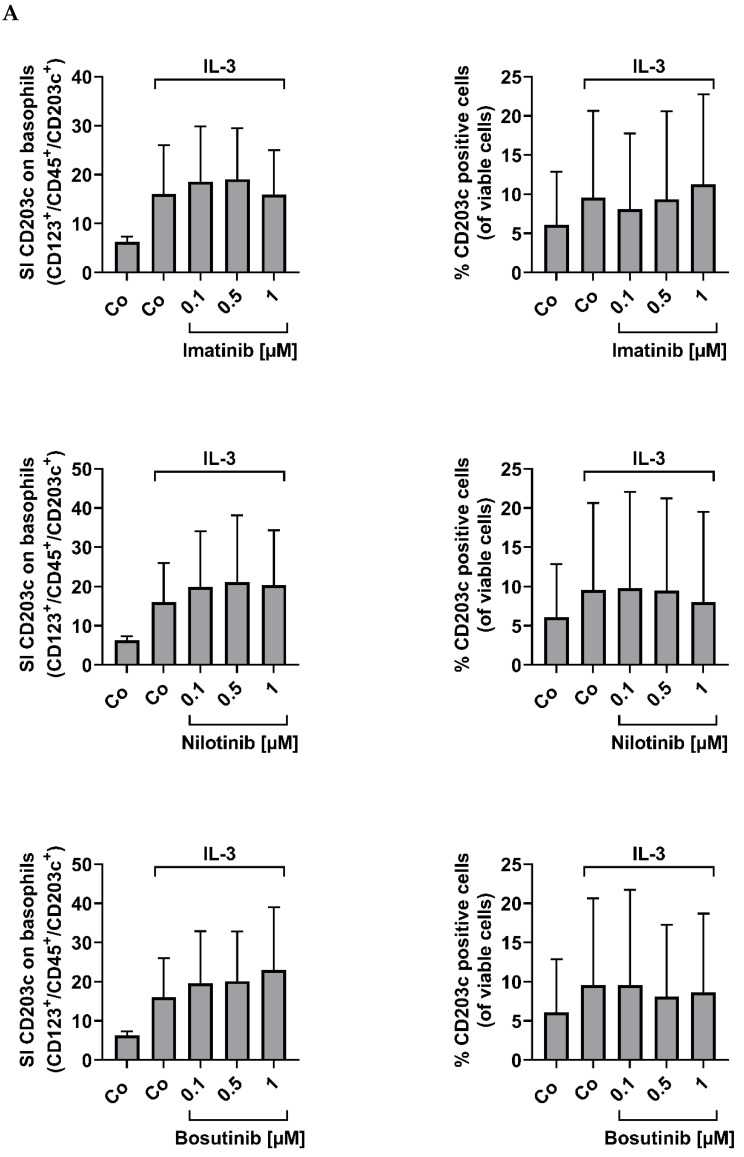

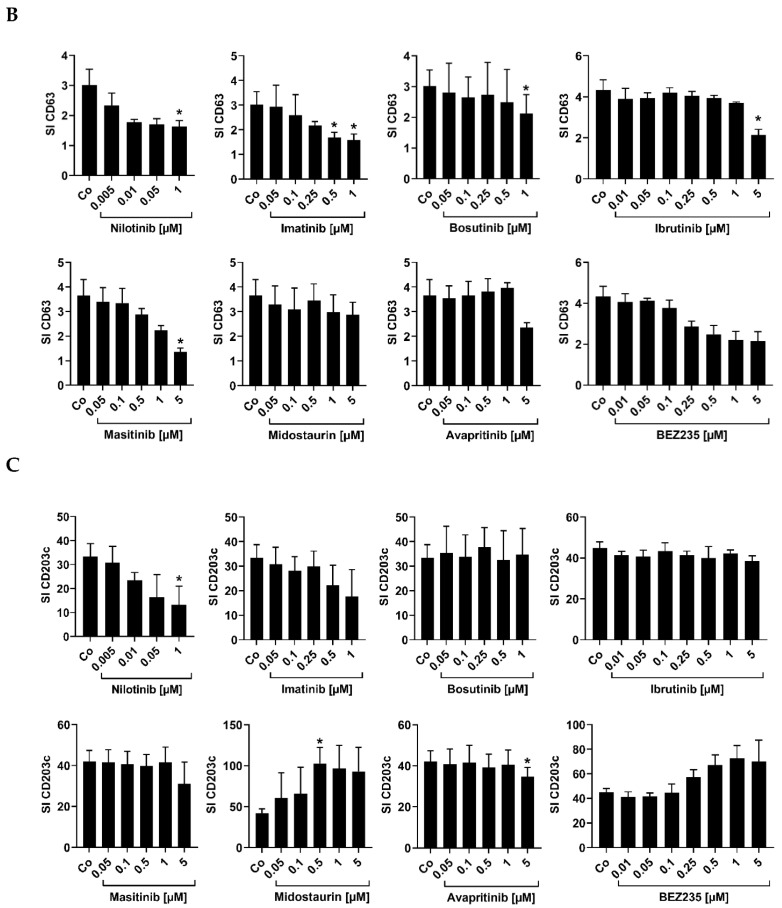
The effect of tyrosine kinase inhibitors on the expression of CD203c and CD63 on KU812 cells and on primary CML basophils. (**A**) Flow cytometry analysis of CD203c expression on peripheral blood (PB) mononuclear cells (MNC) of CML patients after incubation in control medium (Co), medium containing 1 ng/mL IL-3 or IL-3-containing medium and various concentrations of imatinib, nilotinib, or bosutinib (each 0.1, 0.5, or 1 µM) at 37 °C for 48 h. Results are expressed as staining index (SI) of CD203c on basophils (defined as CD123^+^/CD45^+^/CD203c^+^/CD14^−^ cells) (left panels) or percentage (%) of CD203c^+^ cells (right panels). SI was calculated as median fluorescence intensity (MFI) of CD203c on basophils divided by MFI of the isotype control. Results represent the mean ± S.D. from 5 CML donors (#3A, #6A, #18A, #13A, #20A). (**B**,**C**) Flow cytometric evaluation of CD63 (**B**) or CD203c (**C**) expression on KU812 cells after incubation in medium (Co) or medium containing various concentrations of drugs (as indicated) at 37 °C for 48 h. Results are expressed as SI and represent the mean ± S.D. from at least three independent experiments. Asterisk (*): *p* < 0.05 by one-way analysis of variance (ANOVA) with Dunnett’s post hoc test (compared to control).

In patients with CML, basophils reportedly display BCR::ABL1 [32,33]. To define the role of BCR::ABL1 in expression of CD203c, we applied imatinib, nilotinib, and bosutinib. As shown in Figure 4A, incubation with these BCR::ABL1 TKI did not lead to a decrease in CD203c expression on primary CD123^+^/CD203c^+^ CML basophils. Interestingly, imatinib and nilotinib slightly reduced the expression of CD203c and CD63 in the BCR::ABL1^+^ basophil cell line KU812 (Figure 4B,C). However, the other BCR::ABL1-targeting TKI tested did not show inhibitory effects on expression of CD203c on KU812 cells (Figure 4C). These data suggest that basophils in CML express CD203c independent of BCR::ABL1-induced signaling pathways. We also tested the effects of more specific signal transduction inhibitors, including the BTK blocker ibrutinib, the PI3-kinase inhibitors copanlisib and BEZ235, the mTOR inhibitor rapamycin, and the JAK2 blocker fedratinib, on KU812 cells. However, we did not see any substantial effects of these drugs on expression of CD63 or CD203c on KU812 cells (Figure S3).

### 3.5. Cross-Linking of the IgE Receptor on Basophils Is Associated with an Increased Expression of CD203c on CML Basophils

**Figure 5:**
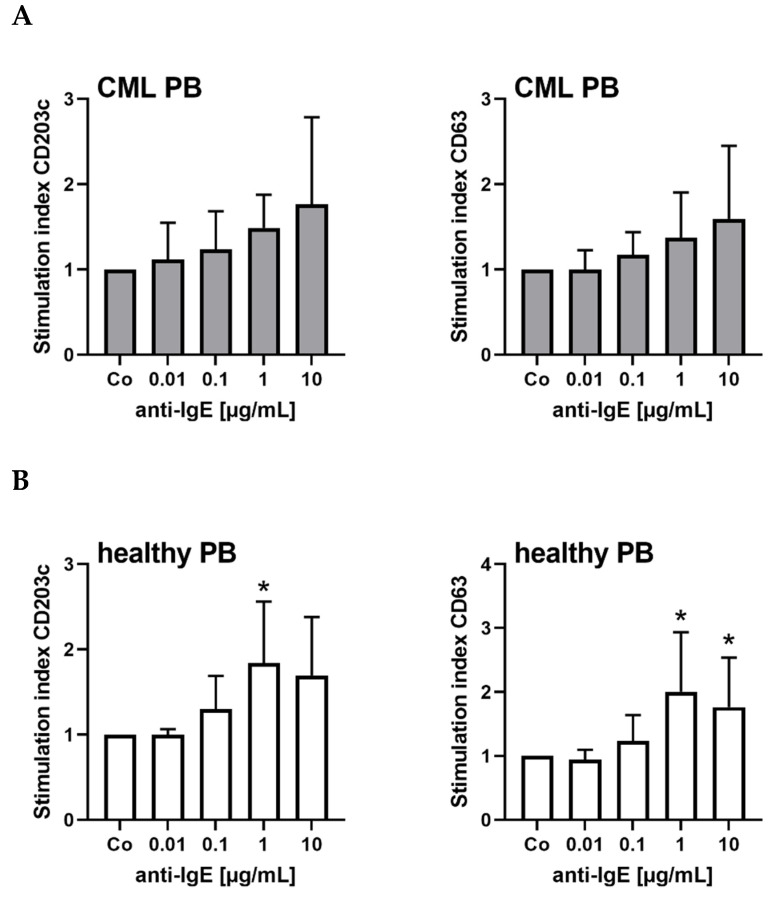
IgE receptor cross-linkage on basophils is associated with upregulation of CD203c and CD63 expression. (**A**) Mononuclear cells (MNC) from six patients with CML (#3A, #6A, #13A, #20A, #28A, #35A; gray bars) and (**B**) seven healthy donors (white bars) were incubated in control medium (Co) or medium containing anti-IgE antibody (0.01–10 µg/mL) at 37 °C for 15 min. Then, cells were examined for expression of CD203c (left panels) and CD63 (right panels) by multi-color flow cytometry. The figures show the stimulation index calculated as mean fluorescence intensities of CD203c or CD63 expression on CD123^+^/CD203c^+^ basophils after anti-IgE stimulation divided by the mean fluorescence intensities of CD203c or CD63 expression on basophils kept in control medium. Results represent the mean ± S.D. from at least six donors. Asterisk (*): *p* < 0.05 as assessed by one way ANOVA with Dunnett’s post hoc test (compared to control).

Cross-linking of the IgE receptor on CML basophils induced by exposure to anti-IgE antibody E124.2.8 was followed by a slight but not significant increase in expression of CD203c (Figure 5A). As expected, the levels of CD63 also increased after activation of the IgE receptor by anti-IgE exposure (Figure 5A). Corresponding results were obtained in normal basophils, confirming the previous literature (Figure 5B) [23,24,25].

### 3.6. The Numbers and Percentage of CD203c^+^ Cells at Diagnosis Correlate with the Risk Category Defined by the SOKAL and EUTOS Scores

**Figure 6:**
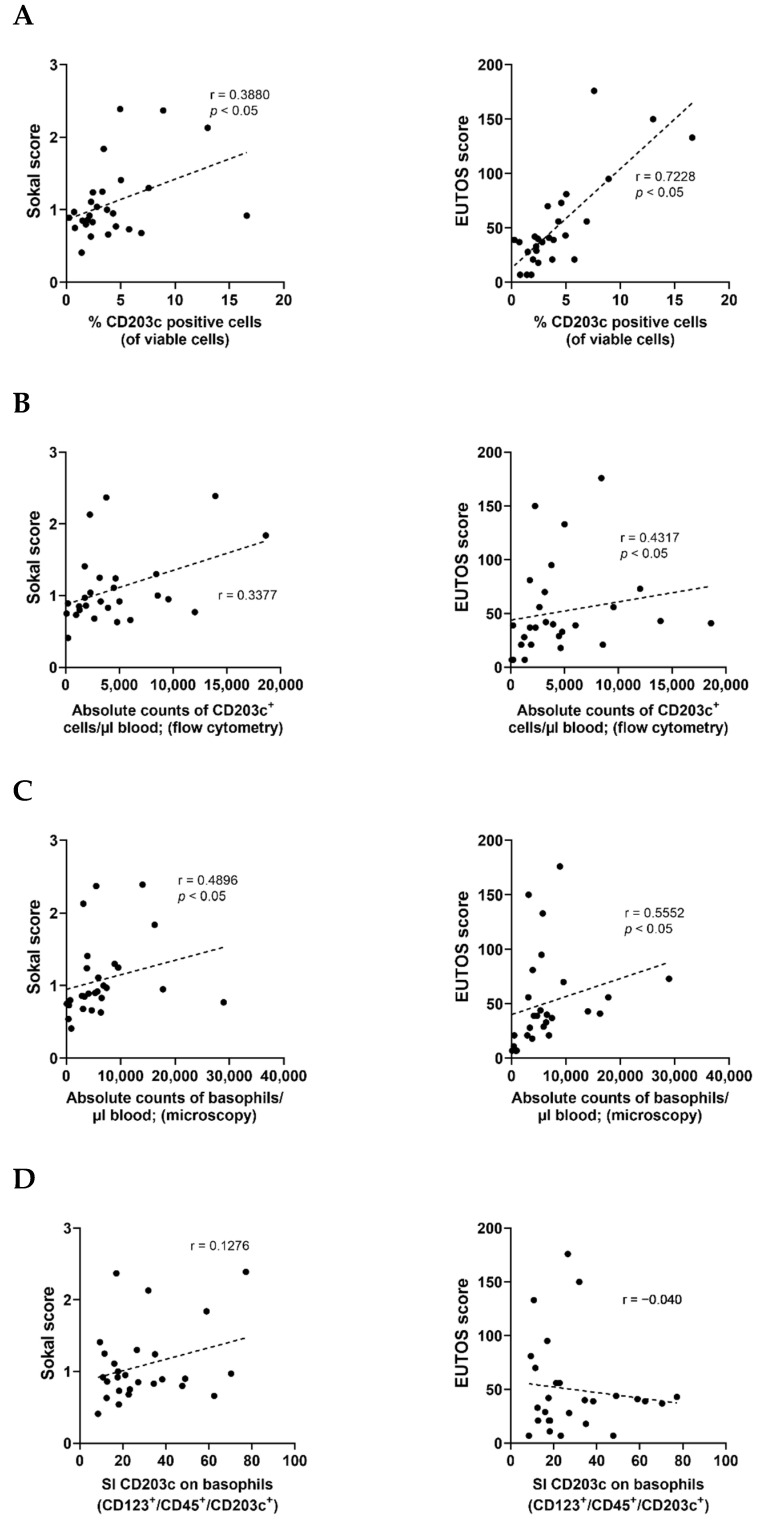

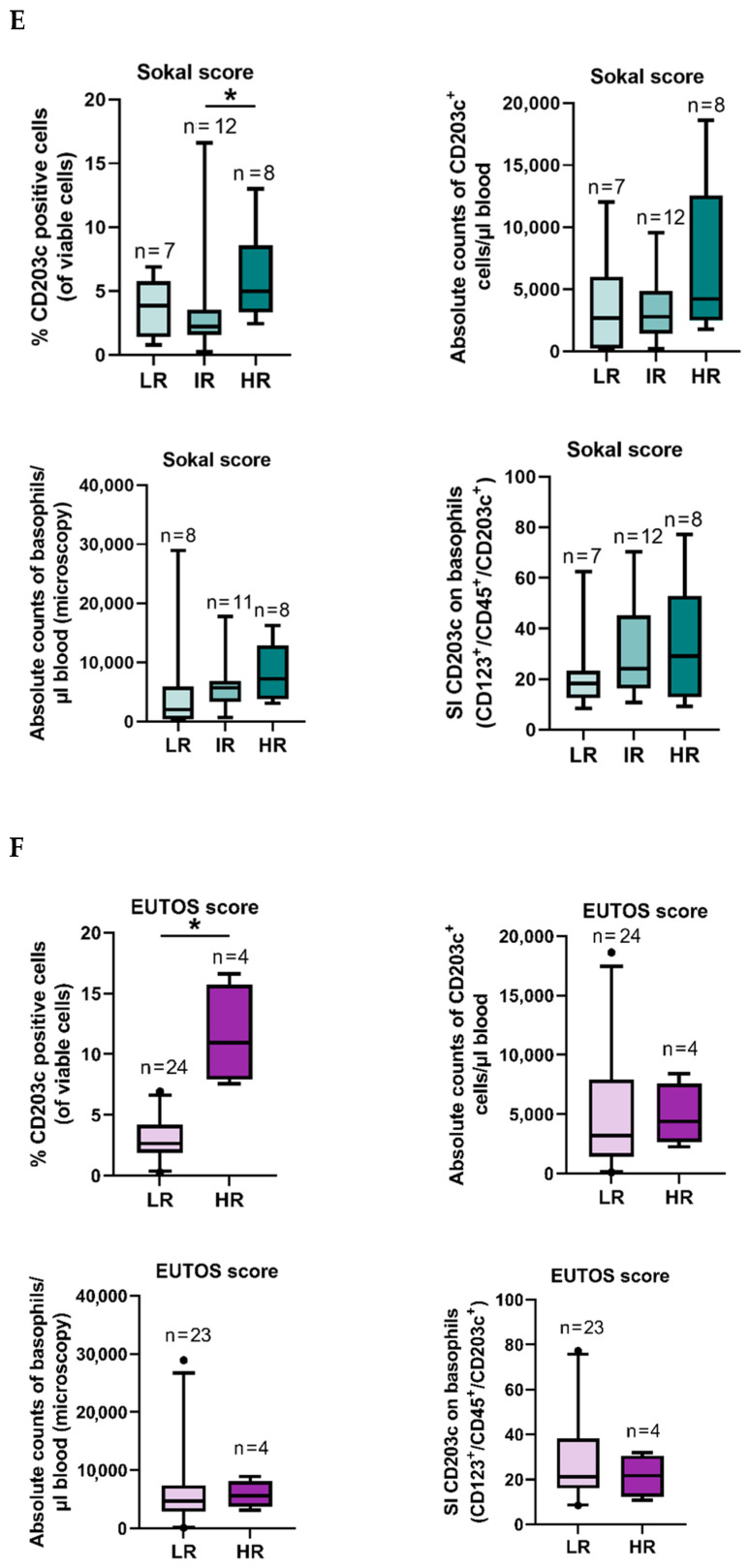
The percentage of CD203c positive cells and the number of basophils roughly correlate with the risk category defined by Sokal and EUTOS scores. (**A**) Correlation of the percentage (%) of CD203c^+^ cells analyzed by flow cytometry with the Sokal score (left panel) or EUTOS score (right panel) in peripheral blood (PB) samples from CML patients at diagnosis (*n* = 27). (**B**) Correlation of the absolute numbers of CD203c^+^ cells determined by flow cytometry with the Sokal score (left panel) or EUTOS score (right panel) in 27 samples from patients with CML. (**C**) Correlation of the absolute numbers of basophils detected by microscopy with the Sokal score (left panel) or EUTOS score (right panel) in PB samples from CML patients at diagnosis (*n* = 27). (**D**) Correlation of the staining index (SI) of CD203c on basophils (defined as CD123^+^/CD45^+^/CD203c^+^/CD14^−^ cells) determined by flow cytometry with the Sokal score (left panel) or EUTOS score (right panel) in CML patients at diagnosis (*n* = 27). (**E**,**F**) The % of CD203c^+^ cells (upper left panel), the absolute count of CD203c^+^ cells (determined using flow cytometry; upper right panel), the absolute basophil count (microscopy; lower left panel), and the staining index (SI) of CD203c on CD123^+^/CD45^+^/CD203c^+^/CD14^−^ basophils (lower right panel) are shown in three different Sokal score risks groups (LR, low-risk; IR, intermediate-risk; HR, high-risk) (shown in different shades of green) (**E**) or in two different EUTOS score risk groups (LR, HR) (shown in different shades of purple). (**F**). The graphs show box and whisker plots (horizontal lines: median values; boxes: 25/75 percentiles; whiskers: 5/95 percentiles; dot: outlier). The SI was calculated using the following formula: median fluorescence intensity (MFI) of CD203c on basophils divided by MFI of respective isotype control. n, numbers of patients analyzed in each group. Abbreviations: *p*, *p*-value; r, Spearman’s rank correlation coefficient. *: *p* < 0.05.

Since basophilia is a most relevant prognostic parameter in CML at diagnosis [13,14,15], we were also interested to know whether the number of CD203c^+^ cells correlate with high-risk CML. We found that the percentage of CD203c^+^ cells correlate with the Sokal score and the EUTOS score in our CML patients (Figure 6A). We also found that the absolute numbers of CD203c^+^ cells correlate with the EUTOS score, but not with the Sokal score (Figure 6B) and that the absolute numbers of basophils (determined by microscopy) correlate with both scoring systems (Figure 6C). The intensity of CD203c expression (staining index) on basophils did not correlate with any risk score (Figure 6D). When comparing the different risk categories (low- until high-risk) in the Sokal and EUTOS scores, we also found significant differences in the percentage of CD203c^+^ cells whereas no substantial differences were seen in the absolute numbers of CD203c^+^ cells, absolute basophil counts, or the CD203c staining index on basophils (Figure 6E,F). Recent data suggest that the numbers of (immature) basophils in CML correlates with basal serum tryptase levels and that tryptase levels at diagnosis are of prognostic significance [19]. However, in the present study, basal serum tryptase levels did not correlate with the numbers or percentages of CD203c^+^ cells (Figure S4).

## 4. Discussion

The ectoenzyme E-NPP3 (CD203c) is a well-known cell surface antigen that is expressed specifically and abundantly in normal blood basophils and is upregulated in response to cross-linking of IgE-binding sites [20,21,22,23,24,25]. Since basophilia is a well-known prognostic feature in CML, we were interested to explore CD203c expression on basophils in our CML patients. In the current study, we show that CD203c is a reliable marker of basophils in CML and that CML basophils display higher levels of CD203c compared to normal basophils. In addition, we found that CD203c levels increase on CML basophils upon cross-linking of IgE receptors in the same way as in normal blood basophils. Finally, we show that the numbers of CD203c^+^ cells decrease during successful TKI therapy in our CML patients.

In normal hematopoiesis, CD203c expression is restricted to basophils, tissue mast cells, and a few myeloid (basophil/mast cells-committed) progenitor cells in the BM [20,21]. In the present study, we show that in patients with CML, the cellular distribution of CD203c is the same as in normal healthy controls. In fact, CD203c was found to be expressed on BM and PB basophils, BM mast cells, and a subset of CD34^+^ progenitor cells in our CML patients. In the PB, CD203c expression appears to be confined to basophils in healthy donors and in patients with CML. In a few patients with CML, however, the percentage of CD203c^+^ cells in the PB was higher than the percentage of basophils assessed by microscopic studies. This observation is best explained by expression of CD203c on circulating basophil-committed progenitor cells in some of these patients, a hypothesis that was confirmed by the observation that CD203c is also detectable on a subset of CD34^+^ progenitor cells in the BM and that these CD203c^+^ basophil-committed progenitor cells are more prevalent in CML patients compared to healthy controls. Finally, some of the premature basophils may also have escaped microscopic examination because the cytoplasm contained only a few specific (basophilic) granules. Expression of CD203c in CML basophils was confirmed by qPCR. In particular, highly purified CML basophils as well as the immature CML-derived basophil cell line KU812 were found to express *CD203c* mRNA. In this regard, it is worth noting that basophils in CML are known to display BCR::ABL1, and thus are clonal cells [32,33].

We also found that the levels of CD203c on basophils are slightly higher in CML compared to healthy donors. Collectively these data suggest that leukemic basophils in CML synthesize more CD203c than normal basophils. However, the mechanisms of overexpression of CD203c on CML basophils remain unknown. One possibility could be that the oncogenic machinery driven by BCR::ABL1 is responsible for expression of CD203c. Indeed, we found that some of the BCR::ABL1-targeting drugs, namely imatinib and nilotinib, downregulate expression of CD203c on KU812 cells. However, bosutinib and asciminib, two other TKI directed against BCR::ABL1, did not downregulate CD203c expression. These data suggest that other signaling pathways and mechanisms are responsible for CD203c expression on basophils. In a next step, we applied several signal-transduction blockers. However, of all drugs tested, including inhibitors of BTK, PI3-kinase, mTOR, KIT, PDGFR, and JAK2, no agent was found to downregulate expression of CD203c on KU812 cells.

Basophilia is one of the most significant prognostic variables in CML [13,14,15]. In the present study we asked whether the numbers of CD203c^+^ cells correlate with basophilia and with other prognostic variables in CML. Indeed, we found that basophil numbers and percentage counts determined by microscopy correlate with the numbers and percentages of CD203c^+^ cells. Moreover, we found a significant correlation of the percentage of CD203c^+^ cells with the Sokal score and the EUTOS score. We also compared basal serum tryptase levels, another prognostic variable related to basophilia, with the numbers and percentages of CD203c^+^ cells. However, no substantial correlation was found which may be explained by the fact that tryptase is synthesized preferentially in immature basophil progenitor cells whereas CD203c is expressed more abundantly in mature basophils. All these data suggest that CD203c is a new biomarker of basophils in CML.

To confirm this hypothesis, we next examined the numbers of CD203c^+^ PB cells during treatment with imatinib and other TKI. We found that the numbers and percentages of CD203c^+^ cells decrease substantially during successful treatment with imatinib and/or other BCR::ABL1-targeting TKI. Moreover, we found a significant correlation between the levels of BCR::ABL1 and the percentage and absolute numbers of CD203c^+^ cells during treatment with imatinib or other BCR::ABL1-targeting TKI. In addition, the levels of CD203c per basophil decreased during therapy, suggesting that most of the basophils detected at the time of MR3 or MR4 are normal basophils.

In normal basophils, CD203c is an established marker of basophil activation that increases transiently during IgE receptor cross-linking [23,24,25]. In the present study, we were able to show that activation of normal basophils and CML basophils through IgE receptor cross-linking is followed by upregulation of CD203c. Interestingly, compared to normal basophils, IgE receptor-dependent upregulation of CD203c on CML basophils was less pronounced which may be explained by the fact that CML basophils are in part more immature cells expressing lower levels of IgE receptors and therefore are less responsive to IgE-dependent activation. Indeed, in all CML patients examined, a substantial subset of basophils was found to display low levels of IgE receptors. Moreover, it is well known that basophils in CML patients are often immature by morphology and may also contain lower amounts of histamine compared to normal basophils. In addition, the oncogenic, BCR::ABL1-induced signaling in CML basophils may produce desensitization and may thereby interfere with optimal signaling through IgE receptors.

The observation that CD203c is a reliable and non-objective quantitative parameter to measure the basophil compartment in CML may have several clinical implications. First, CD203c can be employed as a new prognostic basophil marker in CML, especially when microscopic studies reveal the presence of immature basophils, such as in accelerated phase CML (AP) or in patients with massive left shifting in differential counts. In these patients, CD203c counting may improve and complement microscopic basophil counting. CD203c may also be combined with or integrated in prognostic scores with the aim to improve prognostication. In this regard it is worth noting that basophils have been integrated in most prognostic scoring systems in CML. Finally, CD203c may be included in the diagnostic panel of markers in CML. In fact, although flow cytometry studies are not regarded standard in the initial diagnosis in CML CP, a new robust basophil marker may be a useful tool and support the diagnosis of CML by demonstrating marked or massive basophilia. In some of these cases, excessive basophilia with many immature forms may even lead to the diagnosis of secondary basophilic leukemia [27]. CD203c staining may be a valuable assay to confirm this diagnosis in these patients as also very immature basophils display CD203c.

In summary, CD203c is a new robust marker of basophils in patients with CML. Based on expression of CD203c on mature and immature basophils throughout all stages of differentiation, CD203c is recommended as a pan-basophil marker in these patients and as a potential new flow-marker supporting prognostication in CML. Whether CD203c is able to replace microscopic counting of basophils in the diagnosis or prognostication of CML remains to be determined in forthcoming studies.

## Figures and Tables

**Table 1:** Expression of CD203c on various cell types in patients with CML and in control bone marrow samples.

	Normal PB	Control BM	CML PB	CML BM
**Basophils** CD45^+^/CD123^+^/CD203c^+^/CD14^−^	++	++	++	++
**T cells** CD45^+^/CD3^+^/CD19^−^	−	*−*	−	−
**B cells** CD45^+^/CD19^+^/CD3^−^	−	−	−	−
**Monocytes** CD45^+^/CD14^+^/CD123^−^	−	−	−	−
**NK cells** CD45^+^/CD56^+^/CD3^−^	−	−	−	−
**Neutrophils** CD45^+^/CD16^+^	−	−	−	−
**Eosinophils** CD45^+^/SSC^high^	−	−	−	−
**Mast cells** CD45^+^/CD117^+^/CD34^−^	n.a.	+/− **	n.a.	+/−
**Stem/progenitor cells** CD34^+^/CD45^+^	n.a.	− *	n.a.	− *

CML, chronic myeloid leukemia; PB, peripheral blood; BM, bone marrow; NK, natural killer; CD, cluster of differentiation; SSC, side scatter; n.a., not available. Staining index (SI) = median fluorescence intensity (MFI) of CD203c divided by MFI of matching isotype control. SI scoring system: −, 0–1.3; +/−, 1.31–3; +, 3.01–10; ++, 10.01–100. * a small subpopulation of CD34^+^/CD45^+^ cells expressed CD203c^+^ in patients with CML (3.4–21.7%) and in the normal BM (0.7–1.5%). ** Expression of low levels of CD203c on BM mast cells confirmed our previous data [24,25].

## Data Availability

The data used and/or analyzed during the current study are available from the corresponding author on reasonable request.

## Supplementary Materials

The following are available online at https://www.mdpi.com/article/10.3390/cells12010003/s1. Figure S1: Gating strategy to define basophils in CML samples and healthy donors; Figure S2: Expression of CD203c on immature CML CD34^+^/CD45^+^ stem- and progenitor cells; Figure S3: The effect of tyrosine kinase inhibitors on the expression of CD63 and CD203c on KU812 cells; Figure S4: Correlation of the percentage and absolute numbers of basophils with serum tryptase levels. Table S1A: Patients’ characteristics—CML; Table S1B: Control donors’ characteristics—normal peripheral blood (PB) or control bone marrow (BM); Table S2: Specification of antibodies used in multi-color flow cytometry experiments; Table S3: Expression of *CD203c* mRNA and *CD63* mRNA in sorted basophils and KU812 cells. References [13,15,19,28,29,34,35,36,37,38,39,40,41] are cited in the Supplementary Materials.
