# Supplementary material for: Identification of CD203c as a New Basophil-Specific Flow-Marker in Ph+ Chronic Myeloid Leukemia
Source: Cells. 2022 Dec 20;12(1):3. doi: 10.3390/cells12010003 (PMC9818308; doi:10.3390/cells12010003)
Supplement: Supplementary file 1 [file cells-12-00003-s001.zip › cells-1914392-supplementary.pdf]

Supplementary Materials (Sadovnik et al)
Supplement to Manuscript:

Identification of CD203c as a new basophil-specific flow-marker in Ph^{+} chronic myeloid leukemia

Irina Sadovnik[1,2], Daniel Ivanov[1], Dubravka Smiljkovic[1,2], Gabriele Stefanzl[1,2], Lina Degenfeld-Schonburg[1], Susanne Herndlhofer[1,2], Gregor Eisenwort[1,2,3], Alexander W. Hauswirth[1,2], Thamer Sliwa[3], Felix Keil[2,3], Wolfgang R. Sperr[1,2] and Peter Valent[1,2]

[1]Department of Internal Medicine I, Division of Hematology and Hemostaseology, Medical University of Vienna, Austria; [2]Ludwig Boltzmann Institute for Hematology and Oncology, Medical University of Vienna, Austria; [3]Third Medical Department for Hematology and Oncology, Hanusch Hospital Vienna, Austria.

Materials and Methods

Reagents

Imatinib and nilotinib were purchased from ChemieTek (Indianapolis, IN, USA), bosutinib, masitinib and midostaurin (PKC412) from LC Laboratories (Woburn, MA, USA), avapritinib, BEZ235, copanlisib, fedratinib, and ibrutinib from Selleckchem (Houston, TX, USA), rapamycin from Calbiochem (San Diego, CA, USA) and asciminib (ABL-001) from Active Biochem (Kowloon, Hong Kong). Stock solutions of drugs were prepared by dissolving in dimethyl-sulfoxide (DMSO), 10 % trifluoro acetic acid (copanlisib) (Sigma Aldrich, Saint Louis, MO, USA), or water (imatinib). RPMI 1640 medium and penicillin/streptomycin were purchased from Lonza (Basel, Switzerland), fetal calf serum (FCS) from Gibco/Thermofisher (Waltham, MA, USA) and amphotericin from PAN-Biotech (Aidenbach, Germany). Interleukin 3 (IL-3) was

purchased from PeproTech (London, UK) and anti-IgE from Sigma Aldrich. Ficoll was purchased from Merck (Darmstadt, Germany) and dextran from Leuconostoc spp. from Sigma Aldrich.

Cell lines

The human *BCR::ABL1*+ cell line KU812 was kindly provided by Dr.K.Kishi (Niigata University, Niigata, Japan, 1998). The identity of the cell line was confirmed by the German Collection of Microorganism and Cell Culture (Braunschweig, Germany) using nonaplex-PCR. All experiments in this study were performed from these stocks and cells were kept in culture for a maximum time period of 2 months. KU812 cells were maintained in RPMI 1640 medium supplemented with 10% FCS, 1% penicillin/streptomycin, and 0.5% amphotericin at 37°C.

Patients, diagnostics and follow-up examinations

A total number of 44 patients with CML at diagnosis and/or follow-up (17 females, 27 males) were examined. The median age from our patient cohort at diagnosis was 58 years (range: 26-93 years) and the median age at sampling was 59.5 years (range: 26-93 years). The diagnoses and phase of disease were established according to the World Health Organization (WHO) [1]. Prognostication at diagnosis and risk stratification were performed based on the Sokal score and EUTOS score [2-5]. According to the Sokal score, patients were classified as low-risk (LR; Sokal score <0.8), intermediate-risk (IR; Sokal score 0.8-1.2) and high-risk (HR; Sokal score >1.2). According to the EUTOS score, patients were classified as LR (EUTOS score <87) and HR (EUTOS score ≥87) [4,5]. Bone marrow (BM) and peripheral blood (PB) samples were collected

during routine investigations at diagnosis as well as during follow-up. According to the recommendations of the European Leukemia Net (ELN), karyotyping was performed at diagnosis and in defined time-intervals [5-6]. *BCR::ABL1* mRNA levels were determined in PB and/or BM cells using *ABL1* as a reference gene and the International Scale (IS) for standardized quantification [7-9]. Routine laboratory examinations included BM histology and morphology (BM smears), cytogenetics including FISH from BM samples, molecular studies (BM and PB samples), complete blood counts including differential counts, serum chemistry, and the basal serum tryptase level. Tryptase levels were quantified by a commercial fluoroenzyme-immunoassay as reported [10-12]. The patients´ characteristics are shown in Table S1. Of the 44 patients (CML at diagnosis and/or follow-up), 40 patients had chronic phase CML, 3 had accelerated phase CML, and 1 had blast phase of CML (drug-resistant CML) (Table S1A). Patients were treated with imatinib (400 mg daily per os) and/or other tyrosine kinase inhibitors (TKI) according to ELN guidelines [5,6]. In case of resistance or intolerance against imatinib, patients received a second- or third-line TKI (nilotinib, bosutinib, dasatinib, or ponatinib). In a subset of patients, hydroxyurea was used for initial or palliative cytoreduction. All patients and healthy donors provided written informed consent before PB or BM samples were collected and stored in a local biobank. Basic information about control samples (PB or BM) is provided in Table S1B. Control samples included PB from healthy donors or BM from patients with Non-Hodgkin lymphoma, multiple myeloma and one with Ewing sarcoma. All studies, including storage of cells in a local biobank, drug incubation experiments, and the examination of CML basophils were approved by the local ethics committee of the Medical University of Vienna (approval numbers: 1184/2014, 1063/2018, 1433/2019).

Cell sampling and storage

A total number of 100 cell samples, including 68 samples from 44 patients with CML, control BM samples (n=11) and peripheral blood samples from 21 healthy controls were analyzed in this study. Control BM samples were purchased from Lonza (Basel, Switzerland) (n=5 healthy BM samples) or were obtained from patients with Non-Hodgkin lymphoma (n=2), multiple myeloma (n=3), Ewing sarcoma (n=1), myeloproliferative neoplasms (MPN, n=2), myelodysplastic syndromes (MDS, n=2), chronic myelomonocytic leukemia (CMML, n=2), basophilic leukemia (n=1), and acute myeloid leukemia (AML, n=4). Stabilizer-free heparin (Biochrom AG, Berlin, Germany) was used as anticoagulant. Freshly obtained PB and BM samples were subjected to flow cytometric analysis. Mononuclear cells (MNC) were isolated using Ficoll gradient centrifugation. Isolated MNC were either used immediately for further *in vitro* experiments or were frozen in liquid nitrogen in a local biobank until used. To prevent clumping of cells during thawing, MNC were incubated in RPMI 1640 medium (plus 10% FCS, 1% penicillin/streptomycin, 0.5% amphotericin) supplemented with 100 U/ml DNAse type I (Sigma Aldrich) for 30 minutes at 37°C before used.

Multi-color flow cytometry

Primary CML MNC or KU812 cells were incubated in control medium or medium supplemented with various concentrations of TKI for 48 hours at 37°C. Then, cells were harvested and incubated with a FcR-blocking reagent (Miltenyi Biotec, Bergisch Gladbach, Germany) for 15 minutes to avoid unspecific binding. Thereafter, fluorochrome-labeled mAb were added and incubated in the dark at room temperature

for another 15 minutes. Cells were then washed with phosphate buffered saline (PBS) (Gibco, Gaithersburg, MD, USA), centrifuged, and acquired on a FACSCantoII (BD Biosciences, San José, CA, USA). Flow cytometric analysis was performed to determine the percentage of CD203c$^+$ cells among all viable leukocytes (freshly obtained samples) and the intensity of CD203c expression on basophils in CML samples and control samples (thawed or freshly isolated MNC or whole PB or BM). Fresh PB/BM cells were directly incubated with specific mAb in the dark at room temperature for 15 minutes. MNC were incubated for 15 minutes with FcR-blocking reagent before fluorochrome-labeled mAb were added. After incubation with mAb, freshly obtained cells were further incubated in lysis buffer (BD Biosciences) for 15 minutes. After washing, cells were centrifuged and acquired on a FACSCalibur (BD Biosciences), FACSCantoII (BD Biosciences), or CytoFLEX S (Beckman Coulter, Brea, CA, USA). All flow cytometry data were analyzed using FlowJo software (BD Biosciences). A specification of all mAb used in this study is provided in Table S2.

Purification of CML basophils by cell sorting and quantitative PCR (qPCR)

CML MNC from three patients with CML were isolated using Ficoll. Basophils were enriched by magnetic cell sorting using the basophil isolation kit II (Miltenyi Biotec). Subsequently, CD203c$^+$ cells were purified to homogeneity using a PE-labeled CD203c mAb 97A6 by cell sorting on a FACSAria Fusion (BD Biosciences). Doublets were excluded by their characteristic forward side scatter properties. After cell sorting, the purity of sorted blood basophils was >95%.

To determine mRNA expression levels of *CD203c* and *CD63* in highly purified basophils, qPCR was performed as reported [13] using the following primers: CD203c

forward: CGTTGACTTTAGCAACGGAAC, CD203c reverse: ACATGATCACCAGC AAAGCA, CD63 forward: ACACTGCTTCGATCCT GGAC, CD63 reverse: TCGAC ATGGAAGGGATTTTC. KU812 cells served as a positive-control. Total RNA was extracted from sorted basophils using the RNeasy Plus Micro Kit (Qiagen, Hilden, Germany). *ABL1* served as a reference gene in all analyses (ABL1 forward: TGTATGATTTTGTGGCCAGTGGAG, ABL1 reverse: GCCTAAG ACCCGGAGCT TTTCA).

Supplementary Tables

Table S1A

Patients' characteristics – CML

#	m/f	age [yrs]	D/ FU	CML phase	PB/ BM	WBC [G/L]	PB blasts (%)	BM blasts (%)	Hb (g/dL)	PLT (G/L)	PB baso (%)	Serum tryptase (µg/L)	Karyotype	*BCR::ABL1* % (IS)	Sokal score	EUTOS score	TKI previous	TKI at sampling
1A	m	56	D	CP	PB	153.13	4	1	9.1	382	n.t.	16	46,XY,t(9;22) complex	49.87*	0.92	42	no	no
2A	m	35	D	CP	PB	263	10	2	11.7	124	11	n.t.	n.a.	n.a.**	0.77	73	no	no
3A	m	44	D	AP	PB	17.38	11	19	10.1	567	18	174	46,XY,t(9;22)	69.96	2.13	150	no	no
3B	m	45	FU	n.ap.	PB	7.65	0	n.a.	13.5	302	1	n.t.	n.a.	<0.001	-	-	NIL, IM	NIL
4A	m	33	D	CP	PB	36	0	1	13.8	206	1	7.7	46,XY,t(9;22)	n.a.**	0.54	11	no	no
5A	f	93	D	CP	PB	280.70	6	n.a.	9.8	821	5	n.t.	n.a.	44.03	2.39	43	no	no
6A	m	68	D	CP	PB	228.36	3	1	12.2	140	3	8.3	46,XY,t(9;22)	65.06	1	21	no	no
6B	m	69	FU	n.ap.	PB	8.16	0	n.a.	13.1	133	1	-	n.a.	0.048	-	-	DA	DA
7A	m	60	D	CP	PB	83.73	2	<5	13.5	121	4	n.t.	46,XY,t(9;22)	29.99*	0.85	28	no	no
8A	m	62	D	CP	PB	177	2	1.5	n.t.	n.t.	3	n.t.	46,XY,t(9;22)	n.a.**	0.9	44	no	no
9A	f	61	D	CP	PB	16.84	0	<5	13.3	326	3	n.t.	46,XX,t(9;22)	35.74	0.73	21	no	no
10A	f	30	D	CP	PB	15.7	6	<5	13.4	498	6	n.t.	46,XX,t(9;22)	44.38	0.41	7	no	no
10B	f	32	FU	n.ap.	PB	3.31	0	n.a.	11.3	158	1	<1	n.a.	<0.001	-	-	IM	IM
11A	m	61	D	CP	PB	156.44	1	<5	10.6	275	3	5.7	n.a.	43.19*	0.66	39	no	no
12A	m	37	D	CP	PB	81.56	n.a.	<5	11	309	n.t.	n.t.	n.a.	32.61*	1.04	37	no	no
13A	f	66	D	AP	PB	24.51	4	5	9.2	246	31	18.8	46,XX,t(9;22)	58.929	0.92	133	no	no
13B	f	67	FU	n.ap.	PB	4.43	0	n.a.	10.2	124	1	<1	n.a.	0.003	-	-	IM	IM
13C	f	69	FU	CP	PB	4.85	0	n.a.	9.7	132	2	n.t.	n.a.	2.407	-	-	IM	IM
14A	f	70	D	CP	PB	95.36	2	<5	11.9	867	10	16.6	46,XX,t(9;22)#	50.96	1.25	70	no	no

#	m/f	age [yrs]	D/ FU	CML phase	PB/ BM	WBC [G/L]	PB blasts (%)	BM blasts (%)	Hb (g/dL)	PLT (G/L)	PB baso (%)	Serum tryptase (µg/L)	Karyotype	*BCR::ABL1* % (IS)	Sokal score	EUTOS score	TKI previous	TKI at sampling
14B	f	72	FU	n.ap.	PB	5.52	0	n.a.	11.1	233	1	n.t.	n.a.	<0.001	-	-	IM	IM
15A	f	55	D	CP	PB	541.24	7	<5	7.6	782	3	11.4	46,XX,t(9;22)[#]	34.71	1.84	41	no	no
15B	f	55	FU	CP	PB	5.83	0	n.a.	7.9	207	1	n.t.	46,XX,t(9;22)	36.67	-	-	IM	IM
15C	f	60	FU	n.ap.	PB	7.35	0	n.a.	13.1	271	0	n.t.	n.a.	0.005	-	-	IM, NI, DA	BO
16A	m	63	D	CP	PB	138.62	n.a.	<5	12.6	560	8.8	n.t.	n.a.	n.a.**	n.a.	n.a.	no	no
17B	m	73	FU	CP	PB	3.37	0	n.a.	9.1	213	4	n.t.	n.a.	41.467	-	-	IM	IM
18A	m	71	D	CP	PB	96.06	<1	<5	12.2	320	3	11.2	46,XY,t(9;22)	69.11	0.86	21	no	no
18A	m	71	D	CP	BM	114.61	1	<5	11.2	318	0	13.2	46,XY,t(9;22)	67.982	0.86	21	no	no
18B	m	72	FU	n.ap.	PB	3.26	0	n.a.	12.7	174	1	n.t.	n.a.	0.041	-	-	IM	IM
19A	m	59	D	CP	PB	222.12	1	<5	10.5	789	8	11.1	46,XY,t(9;22)[#]	39.42*	0.95	56	no	no
19B	m	64	FU	CP	PB	8.79	0	n.a.	12.8	348	1	-	n.a.	7.027 T315I	-	-	IM, DA, BO	PO
20A	m	80	D	CP	PB	111.25	<1	1	9.1	801	8	13.3	46,XY,t(9;22)[#]	68.98	1.3	176	no	no
20B	m	81	FU	n.ap.	PB	10.12	0	n.a.	6.9	336	0	8.4	n.a.	0.117	-	-	IM	DA
20C	m	83	FU	CP	PB	10.66	0	n.a.	15.1	436	10	n.t.	n.a.	24.33	-	-	IM, DA	BO
21A	f	53	D	CP	PB	42.4	5	<5	9.9	1635	13	36.2	46,XX,t(9;22)[#]	34.95	2.37	95	no	no
21B	f	54	FU	n.ap.	PB	5.65	0	n.a.	11.6	222	0	n.t.	n.a.	0.13	-	-	IM	IM
22A	f	57	D	CP	PB	35.24	1	<5	11.5	1378	11	2.5	46,XX,t(9;22)	51.03	1.41	81	no	no
23A	m	63	D	CP	PB	72.11	1	<5	12.9	320	1	12.3	45,X,-Y,t(9;22)	39.993	0.8	7	no	no
23B	m	68	FU	n.ap.	PB	8.25	0	n.a.	13.6	378	0	-	n.a.	0.008	-	-	IM	IM
24A	m	71	D	CP	PB	189.61	4	1	9.2	206	2	19.8	46,XY,t(9;22)	39.69	1.24	18	no	no
24B	m	72	FU	CP	PB	6.53	0	n.a.	11.1	234	0	n.t.	n.a.	2.055	-	-	IM	IM
25A	m	59	D	CP	PB	247.61	2	<5	11.2	282	3	18.7	46,XY,t(9;22)	43.08	0.97	37	no	no
25B	m	63	FU	n.ap.	PB	7.58	0	n.a.	12.3	228	1	n.t.	n.a.	0.162	-	-	IM, NI	IM
25C	m	65	FU	n.ap.	PB	10.63	0	n.a.	11.9	235	0	n.t.	n.a.	0.017	-	-	IM, NI	IM

#	m/f	age [yrs]	D/ FU	CML phase	PB/ BM	WBC [G/L]	PB blasts (%)	BM blasts (%)	Hb (g/dL)	PLT (G/L)	PB baso (%)	Serum tryptase (µg/L)	Karyotype	*BCR::ABL1* % (IS)	Sokal score	EUTOS score	TKI previous	TKI at sampling
26A	m	65	D	CP	PB	11.53	n.t.	<5	13.4	213	1	7.9	46,XY,t(9;22)	26.96*	0.75	7	no	no
27A	m	78	D	CP	PB	196.47	2	<5	11.7	253	3	19.1	46,XY,t(9;22)	53.79	1.11	29	no	no
27B	m	79	FU	n.ap.	PB	4.42	0	n.a.	11.8	117	1	n.t.	n.a.	0.192	-	-	IM	IM
28A	f	40	D	CP	PB	81.99	1	<5	12.7	960	5	16.7	46,XX,t(9;22)[◊]	33.67	0.89	39	no	no
28A	f	40	D	CP	BM	73.86	0	3	12.8	940	3	15.5	46,XX,t(9;22)[◊]	44.585	0.89	39	no	no
28B	f	41	FU	n.ap.	PB	5.9	0	n.a.	12.7	209	1	4.5	n.a.	0.008	-	-	NI	NI
29A	f	33	D	CP	PB	38.69	1	<5	13.2	732	8	9.3	46,XX,t(9;22)	25.68	0.68	56	no	no
30A	m	26	D	CP	PB	211.53	2	<5	7.5	224	3	6.4	46,XY,t(9;22)[◊]	49.10	0.63	33	no	no
30B	m	29	FU	n.ap.	PB	5.68	0	n.a.	15.8	168	1	n.t.	n.a.	0.269	-	-	IM	IM
31A	m	51	D	CP	PB	162.2	2	<5	16.4	164	4	17.6	46,XY,t(9;22)	43.45	0.83	40	no	no
31B	m	56	FU	n.ap.	PB	8.04	0	n.a.	15.5	222	1	n.a.	n.a.	0.004	-	-	IM, NI	IM, NI
32B	f	51	FU	n.ap.	PB	5.46	0	n.a.	13.4	272	1	n.t.	46,XX	0.008	-	-	IM	IM
33B	f	59	FU	n.ap.	PB	3.55	0	n.a.	11	212	1	n.t.	n.a.	<0.001	-	-	IM, DA	IM, DA
34B	f	78	FU	n.ap.	PB	7.48	0	n.a.	10	208	1	n.t.	46,XX	<0.001	-	-	IM, NI	BO
35A	m	27	D	CP	PB	285.56	<1	<5	7.8	167	2	n.t.	46,XY,t(9;22)[◊]	39.179	0.51	14	no	no
35B	m	27	FU	CP	PB	8.47	0	n.a.	12.7	46	0	12	n.a.	35.21	-	-	IM	IM
36B	f	29	FU	BP	PB	13.99	43	n.a.	10.5	299	8	n.t.	n.a.	46.64	-	-	IM, DA	DA
37A	f	79	D	CP	BM	40.1	1	0.3	11.9	350	7	n.t.	n.a.	78*	n.a.	n.a.	no	no
38A	m	58	D	CP	BM	15.16	0	1	14.3	629	5	n.t.	46,XY, t(9;22), del(9), der(22)	38.67*	0.8	35	no	no
39A	f	49	D	CP	BM	95.4	0	0.3	11.8	398	2	n.t.	46,XY,t(9;22)[◊]	85*	n.a.	n.a.	no	no
40A	m	40	D	CP	BM	13.0	0	1	13.9	601	1	n.t.	46,XY,t(9;22)	46.4	n.a.	n.a.	no	no
41A	m	39	D	AP	BM	190.9	3	2	11	347	15	n.t.	46,XY,t(9;22), t(8;17)	56.38	n.a.	n.a.	no	no
42A	m	62	D	CP	PB	31.43	2	<5	13.1	869	11	7.4	46,XY,t(9;22)	69.25	n.a.	n.a.	no	no

#	m/f	age [yrs]	D/ FU	CML phase	PB/ BM	WBC [G/L]	PB blasts (%)	BM blasts (%)	Hb (g/dL)	PLT (G/L)	PB baso (%)	Serum tryptase (µg/L)	Karyotype	***BCR::ABL1*** % (IS)	Sokal score	EUTOS score	TKI previous	TKI at sampling
43A	f	58	D	CP	PB	57.32	<1	1	14.1	465	7	n.t.	n.a.	72.358	n.a.	n.a.	no	no
44A	m	33	D	CP	BM	439.1	4.4	1.5	8.5	699	15	n.t.	n.a.	97*	n.a.	n.a.	no	no

The patients´ characteristics were obtained at diagnosis (A) and/or during follow-up (B,C). All follow-up patients were treated with a *BCR::ABL1* tyrosine kinase inhibitor (TKI) for at least 1 month. Abbreviation: #, patient number; yrs, years; m, male; f, female; D, diagnosis, FU, follow-up; PB, peripheral blood; BM, bone marrow; WBC, white blood count; G/L, 10^9 cells per liter; Hb, hemoglobin; g/dL, gram per deciliter; PLT, platelet count; baso, basophils; µg/L, microgram per liter; IS, international scale; CP, chronic phase; AP, accelerated phase; BP, blast phase; IM, imatinib; NIL, nilotinib; DA, dasatinib; BO, bosutinib; PO, ponatinib; ◊, in these patients, the karyotype was established by fluorescence in situ hybridization (FISH) only but not by conventional karyotyping, in all other patients, the karyotype was also determined by conventional cytogenetic analysis; *, *BCR::ABL1* mRNA levels were measured by qPCR in BM cells and were expressed as percent of *ABL1* mRNA and adjusted according to the international scale (IS); n.a.**, in these patients, the presence of *BCR::ABL1* mRNA was confirmed by PCR but was not quantified (at the time of analysis no quantitative PCR was available); -, data not available for follow-up samples; n.t.; not tested; n.a., not available; n.ap., not applicable.

Table S1B

Control donors´ characteristics–normal peripheral blood (PB) or control bone marrow (BM)

#	m/f	age [yrs]	PB/BM	WBC [G/L]	Diagnosis
1	m	34	PB	n.a.	n.ap.
2	f	34	PB	6.25	n.ap.
3	f	28	PB	7.59	n.ap.
4	f	26	PB	n.a.	n.ap.
5	f	26	PB	n.a.	n.ap.
6	f	36	PB	n.a.	n.ap.
7	f	29	PB	n.a.	n.ap.
8	m	24	PB	n.a.	n.ap.
9	m	38	PB	6.6	n.ap.
10	m	37	PB	n.a.	n.ap.
11	m	51	PB	n.a.	n.ap.
12	m	27	PB	6.4	n.ap.
13	m	34	PB	5.25	n.ap.
14	m	26	PB	n.a.	n.ap.
15	f	41	PB	5.7	n.ap.
16	f	26	PB	5.03	n.ap.
17	f	34	PB	14.5	n.ap.
18	m	29	PB	6.2	n.ap.
19	f	41	PB	8.76	n.ap.
20	m	52	PB	n.a.	n.ap.
21	f	41	PB	7.7	n.ap.
22	n.a.	n.a.	BM	n.a.	Normal BM purchased from Lonza (Donor: 2810C)
23	n.a.	n.a.	BM	n.a.	Normal BM purchased from Lonza (Donor: 3290B)
24	n.a.	n.a.	BM	n.a.	Normal BM purchased from Lonza (Sample: 0000533233)
25	f	35	BM	7.7	suspected hematologic neoplasm; Ewing sarcoma*
26	f	51	BM	7.4	NHL*
27	m	73	BM	3.06	NHL*
28	n.a.	n.a.	BM	n.a.	Normal BM purchased from Lonza (Sample: 18TL331637)
29	n.a.	n.a.	BM	n.a.	Normal BM purchased from Lonza (Donor: 44009)
30	m	82	BM	15.37	MM
31	f	72	BM	8.91	MM
32	f	72	BM	3.71	MM

33	m	n.a.	BM	64	CMML-0
34	m	n.a.	PB	79	CMML-0
35	m	40	PB	66	MPN-PMF
36	m	70	BM	2.89	AML
37	m	4.77	BM	85	AML
38	m	2.48	BM	63	MDS
39	m	1.60	BM	63	AML
40	f	7.40	BM	70	MPN-PMF
41	m	27.25	BM	46	Basophilic Leukemia
42	m	15.94	BM	69	AML
43	f	7.65	BM	88	MDS

Abbreviation: #, patient number; m, male; f, female; yrs, years; PB, peripheral blood; BM, bone marrow; WBC, white blood count; G/L, 10^9 cells per liter; n.a., not available; n.ap., not applicable; NHL, Non-Hodgkin lymphoma; MM, multiple myeloma; CMML, chronic myelomonocytic leukemia; MPN, myeloproliferative neoplasm; PMF, primary myelofibrosis; AML, acute myeloid leukemia; MDS, myelodysplastic syndrome. *, no infiltration with neoplastic cells was found.

Table S2

Specification of antibodies used in multi-color flow cytometry experiments

CD	Antigen	Clone	Conjugate	Species, Isotype	Manufacturer
n.c.	isotype control	MOPC-21	PE	Mouse, IgG1	BD Biosciences
n.c.	isotype control	MPC-11	APC-Cy7	Mouse, IgG2b	BioLegend
CD3	TcR	UCHT1	APC	Mouse, IgG1	BD Biosciences
CD14	LPSR	TÜK4	FITC	Mouse, IgG2A	Dako
CD16	FcγRIII	CLB/FcGran1	FITC	Mouse, IgG2A	BD Biosciences
CD19	B4	4G7	FITC	Mouse, IgG1	BD Biosciences
CD34	HPCA-1	581	Pacific blue	Mouse, IgG1	BioLegend
CD45	LCA	HI30	V500	Mouse, IgG1	BD Biosciences
CD45	LCA	2D1	PerCP	Mouse, IgG1	BD Biosciences
CD56	NCAM	MEM-188	FITC	Mouse, IgG2A	BioLegend
CD63	LAMP-3	CLBGran/12	FITC	Mouse, IgG1	Beckman Coulter
CD117	SCFR/KIT	104D2	PE-Cy7	Mouse, IgG1	Invitrogen
CD123	IL-3RA	AC145	APC	Mouse, IgG2A	Miltenyi Biotec
CD203c	E-NPP3	97A6	PE	Mouse, IgG1	Beckman Coulter
n.c.	FcεRIα	AER-37 (CRA-1)	APC-Cy7	Mouse, IgG2b	BioLegend

Abbreviation: CD, cluster of differentiation; PE, phycoerythrin; FITC, fluorescein isothiocyanate; PerCP, peridinin chlorophyll protein; APC, allophycocyanin; Cy7, cyanine7; n.c., not (yet) clustered; TcR, T cell receptor; LPSR, lipopolysaccharide-related antigen; FcγRIII, Fc gamma receptor III; HPCA-1, human precursor cell antigen-1; LCA, leukocyte common antigen; NCAM, neural cell adhesion molecule; LAMP-3; lysosomal-associated membrane protein 3; SCFR, stem cell factor receptor; IL-3RA, interleukin 3 receptor alpha; E-NPP3; ectonucleotide pyrophosphatase/ phosphodiesterase 3; FcεRIα, Fc epsilon receptor I alpha.
Company Locations: BD Biosciences, San José, CA, USA; Dako, Glostrup, Denmark; Miltenyi Biotec, Bergisch Gladbach, Germany; BioLegend, San Diego, CA, USA; Beckman Coulter, Brea, CA, USA; Invitrogen, Carlsbad, CA, USA.

Table S3

Expression of *CD203c* mRNA and *CD63* mRNA in sorted basophils and KU812 cells

Patient/ Cell line	Phase of CML	Cell type	Expression of mRNA for* *CD203c*	*CD63*
patient #13A	AP	purified basophils	+/–	++
patient #18A	CP	purified basophils	+	+++
patient #35A	CP	purified basophils	+	+++
KU812**	-	basophil CML cell line**	+	+++

Primary CD203c^{+} blood basophils were purified to homogeneity (>95%) from 3 patients with CML by flow cytometry and cell sorting. In sorting experiments, cell doublets were excluded by their forward side scatter properties. RNA was isolated from these highly purified CML basophils or KU812 cells and analyzed for expression of *CD203c* mRNA and *CD63* mRNA expression using qPCR. Technical details and the sequences of the PCR primers used are provided in the section Materials and Methods of this Supplement. *The following score was used to define expression levels of *CD63* and *CD203c* mRNA: –, 0-10% expression relative to *ABL1* mRNA; +/–, 11-100% expression relative to *ABL1* mRNA; +, 101-500% expression relative to *ABL1* mRNA; ++, 501-1,000% expression relative to *ABL1* mRNA; +++, >1,000% expression relative to *ABL1* mRNA. **The basophilic cell line KU812 was grown from a patient with CML and contains the BCR::ABL1 oncoprotein. #, patient numbers refer to numbers in Table S1A; CML, chronic myeloid leukemia; AP, accelerated phase; CP, chronic phase.

Supplementary Figures

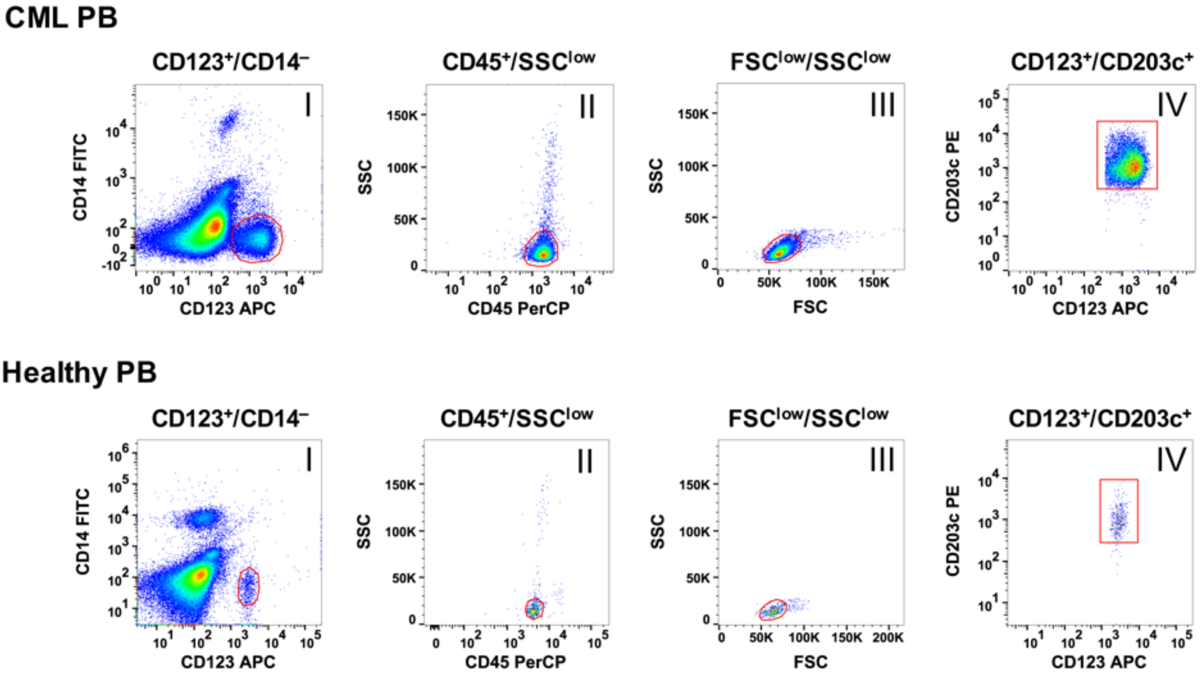

Sadovnik et al., Supplementary Figure S1

Gating strategy to define basophils in CML samples and healthy donors

The gating strategy for the identification of CD123$^+$/CD45$^+$/CD203c$^+$/CD14$^–$ basophils is shown in an example of a peripheral blood sample obtained from a CML patient in CP (upper panel, #21A) and a blood sample of a healthy donor (lower panel, #11). Multicolor flow cytometry was performed on a FACSCantoII and analyzed using FlowJo software. In both samples, a sequential gating strategy (steps I-IV) was applied to identify basophils. In a first step, the basophil-containing fraction was defined by expression of CD123 and exclusion of CD14$^+$ cells (I). Then, cells were gated as CD45$^+$/SSC^{low} cells to exclude SSC^{high} (non-basophil) cells (II). Thereafter, viable cells were selected by their light-scatter properties (III). Based on the corresponding isotype control antibody for CD203c$^+$ (data not shown), the final gate was set to identify the CD123$^+$/CD203c$^+$ population (IV) which was further analyzed. #, patient/donor numbers refer to numbers in Table S1A and S1B.

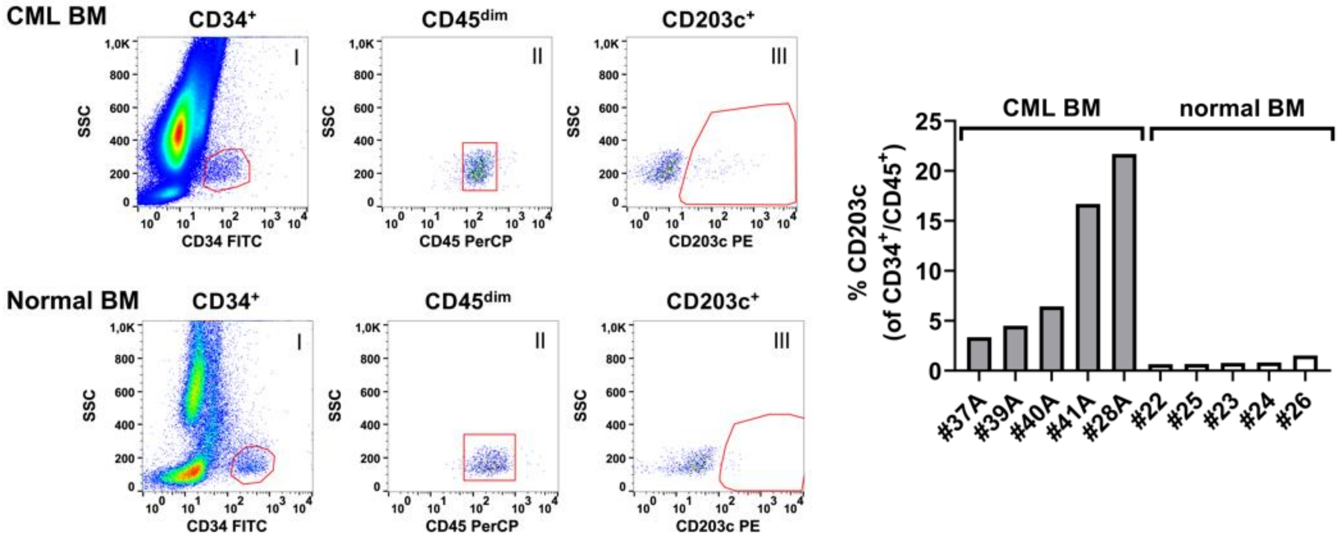

Sadovnik et al., Supplementary Figure S2

Expression of CD203c on immature CML CD34$^+$/CD45$^+$ stem- and progenitor cells

Left panel: bone marrow (BM) cells from a patient with CML (upper panels; #40A) and a normal BM control (lower panels; #26) are shown in the dot plots. Cells were analyzed by multi-color flow cytometry using a FACSCantoII (BD Biosciences). After gating for CD34$^+$ cells (gate I), cells were further defined by CD45^{dim} (gate II). Finally, the expression of CD203c (gate III) was determined on immature CD34$^+$/CD45$^+$ stem- and progenitor cells. The gate for CD203c was set based on the expression of the matching isotype control antibody (not shown). Right panel: the bar diagram shows the percentage of CD203c of CD34$^+$/CD45$^+$ cells in BM samples from 5 patients with CML (grey bars) and 5 normal BM controls (white bars). #, patient/donor numbers refer to numbers in Table S1A and S1B.

A

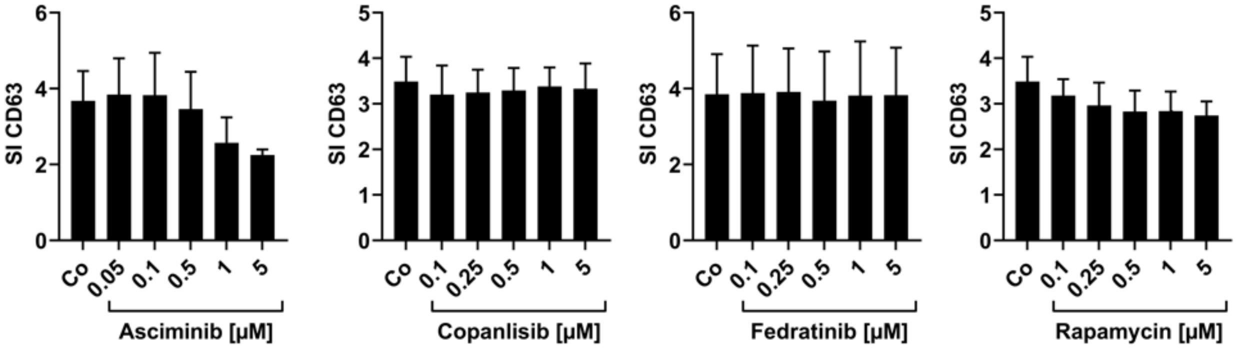

B

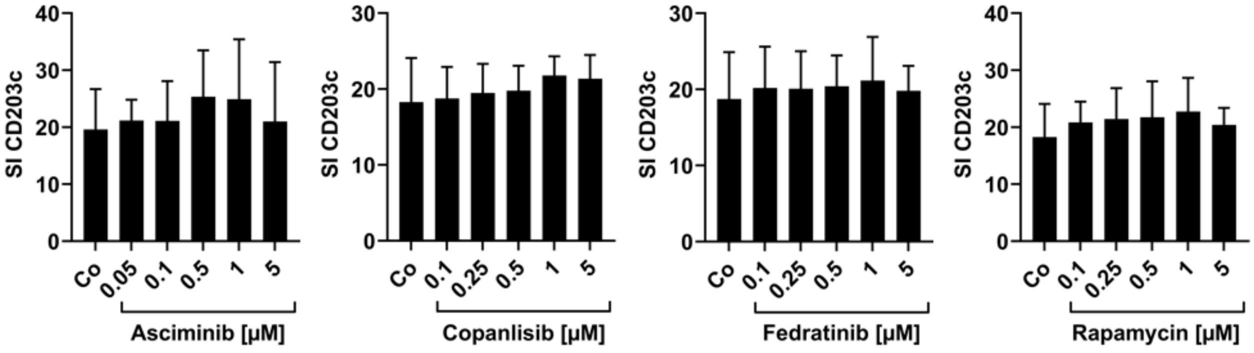

Sadovnik et al., Supplementary Figure S3

The effect of tyrosine kinase inhibitors on the expression of CD63 and CD203c on KU812 cells

Flow cytometric evaluation of CD63 (**A**) or CD203c (**B**) expression on KU812 cells after incubation in medium (Co) or medium containing various concentrations of different compounds (as indicated) at 37°C for 48 hours. Results are expressed as staining index (SI = mean fluorescence intensity of CD63 or CD203c divided by mean fluorescence intensity of respective isotype control) and represent the mean±S.D. from 3 independent experiments. DMSO was used as a solvent control and showed no effect (data not shown).

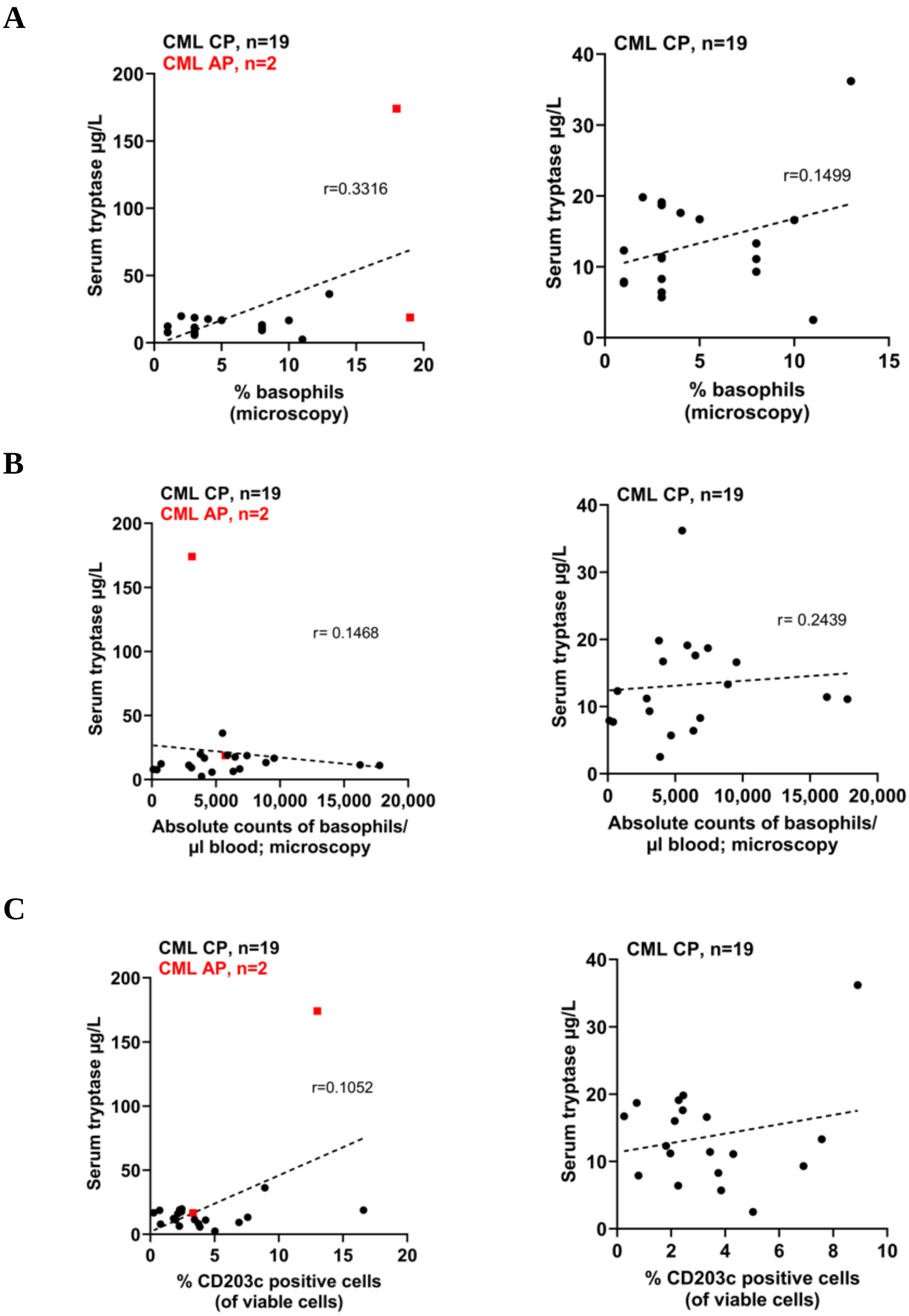
A
CML CP, n=19
CML AP, n=2
Serum tryptase µg/L
r=0.3316
% basophils
(microscopy)
CML CP, n=19
r=0.1499
B
CML CP, n=19
CML AP, n=2
r= 0.1468
Absolute counts of basophils/
µl blood; microscopy
CML CP, n=19
r= 0.2439
C
CML CP, n=19
CML AP, n=2
r=0.1052
% CD203c positive cells
(of viable cells)
CML CP, n=19

D

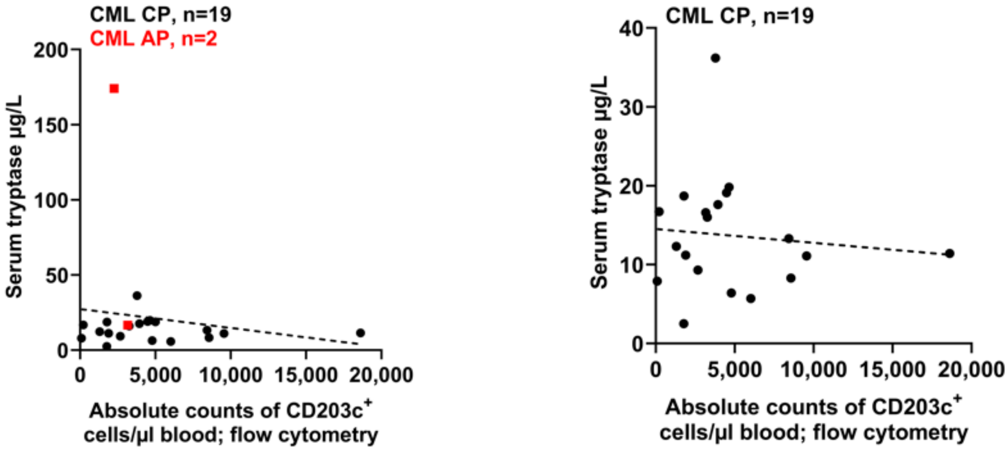

Sadovnik et al., Supplementary Figure S4

Correlation of the percentage and absolute numbers of basophils with serum tryptase levels

(**A**) Correlations of serum tryptase levels (µg/L) with the percentage (%) of basophils determined using microscopy are shown in peripheral blood (PB) samples from patients with CML at diagnosis (left panel: CML CP, n=19; CML AP, n=2; right panel: CML CP, n=19). **(B)** Correlation of serum tryptase levels with the absolute numbers of basophils detected by microscopy/µl blood are shown in 19 samples from CML CP patients (left and right panel) and 2 samples from CML AP patients (left panel). (**C**) Correlations of serum tryptase levels with the percentage of CD203c$^+$ cells determined using flow cytometry are shown in 19 PB samples from patients with CML CP (left and right panel) and 2 samples from patients with CML AP (left panel). **(D)** Correlation of serum tryptase levels with the absolute numbers of CD203c$^+$ cells/µl blood detected by flow cytometry are shown (left panel: CML CP, n=19; CML AP, n=2; right panel: CML CP, n=19). The red squares in the diagrams represent the AP samples, while the black circles represent CML CP samples. To calculate the absolute count of basophils or absolute count of CD203c$^+$ cells per µl blood following formula was used: % basophils (microscopy) or CD203$^+$ cells (flow cytometry)/100*WBC [G/L]*1000. r, Spearman's rank correlation coefficient; CP, chronic phase; AP, accelerated phase; CML, chronic myeloid leukemia; µg, microgram; L, liter.

References

1. Vardiman, J.W.; Melo, J.; Baccarani, M.; Thiele, J. Chronic myelogenous leukemia, BCR/ABL1 positive. In: Swerdlow, S.H.; Campo, E.; Harris, N.L.; et al, editors. WHO Classification of Tumours of Haematopoietic and Lymphoid Tissues, 4th Edition. Lyon, France: IARC Press, **2008**, pp. 32-37.

2. Sokal, J.E.; Cox, E.B.; Baccarani, M.; Tura, S.; Gomez, G.A.; Robertson, J.E.; Tso, C.Y.; Braun, T.J.; Clarkson, B.D.; Cervantes, F.; et al. Prognostic discrimination in "good-risk" chronic granulocytic leukemia. *Blood* **1984**, *63*, 789-799.

3. Hasford, J.; Pfirrmann, M.; Hehlmann, R.; Allan, N.C.; Baccarani, M.; Kluin-Nelemans, J.C.; Alimena, G.; Steegmann, J.L.; Ansari, H. A new prognostic score for survival of patients with chronic myeloid leukemia treated with interferon alfa. Writing Committee for the Collaborative CML Prognostic Factors Project Group. *J. Natl. Cancer Inst.* **1998**, *90*, 850-858.

4. Hasford, J.; Baccarani, M.; Hoffmann, V.; Guilhot, J.; Saussele, S.; Rosti, G.; Guilhot, F.; Porkka, K.; Ossenkoppele, G.; Lindoerfer, D.; et al. Predicting complete cytogenetic response and subsequent progression-free survival in 2060 patients with CML on imatinib treatment: the EUTOS score. *Blood* **2011**, *118*, 686-692.

5. Baccarani, M.; Saglio, G.; Goldman, J.; Hochhaus, A.; Simonsson, B.; Appelbaum, F.; Apperley, J.; Cervantes, F.; Cortes, J.; Deininger, M.; et al; European LeukemiaNet. Evolving concepts in the management of chronic myeloid leukemia: recommendations from an expert panel on behalf of the European LeukemiaNet. *Blood* **2006**, *108*, 1809-1820.

6. Baccarani, M.; Cortes, J.; Pane, F., Niederwieser, D.; Saglio, G.; Apperley, J.; Cervantes, F.; Deininger, M.; Gratwohl, A.; Guilhot, F.; et al; European LeukemiaNet. Chronic myeloid leukemia: an update of concepts and management recommendations of European LeukemiaNet. *J. Clin. Oncol.* **2009**, *27*, 6041-6051.

7. Martinelli, G.; Iacobucci, I.; Soverini, S.; Cilloni, D.; Saglio, G.; Pane, F.; Baccarani, M. Monitoring minimal residual disease and controlling drug resistance in chronic myeloid leukaemia patients in treatment with imatinib as a guide to clinical management. *Hematol. Oncol.* **2006**, *24*, 196-204.

8. Hughes, T.; Deininger, M.; Hochhaus, A.; Branford, S.; Radich, J.; Kaeda, J.; Baccarani, M.; Cortes, J.; Cross, N.C.; Druker, B.J.; et al. Monitoring CML patients responding to treatment with tyrosine kinase inhibitors: review and recommendations for harmonizing current methodology for detecting BCR-ABL transcripts and kinase domain mutations and for expressing results. *Blood* **2006**, *108*, 28-37.

9. Müller, M.C.; Cross, N.C.; Erben, P.; Schenk, T.; Hanfstein, B.; Ernst, T.; Hehlmann, R.; Branford, S.; Saglio, G.; Hochhaus, A. Harmonization of molecular monitoring of CML therapy in Europe. *Leukemia* **2009**, *23*, 1957-1963.

10. Sperr, W.R.; Pfeiffer, T.; Hoermann, G.; Herndlhofer, S.; Sillaber, C.; Mannhalter, C.; Kundi, M.; Valent, P. Serum-tryptase at diagnosis: a novel biomarker improving prognostication in Ph(+) CML. *Am. J. Cancer Res.* **2014**, *5*, 354-362.

11. Sperr, W.R.; El-Samahi, A.; Kundi, M.; Girschikofsky, M.; Winkler, S.; Lutz, D.; Endler, G.; Rumpold, H.; Agis, H.; Sillaber, C.; et al. Elevated tryptase levels selectively cluster in myeloid neoplasms: a novel diagnostic approach and screen marker in clinical haematology. *Eur. J. Clin. Invest.* **2009**, *39*, 914-923.

12. Valent, P.; Sperr, W.R.; Sotlar, K.; Reiter, A.; Akin, C.; Gotlib, J.; Horny, H.P.; Arock, M. The serum tryptase test: an emerging robust biomarker in clinical hematology. *Expert Rev. Hematol*. **2014**, *7*, 683-690.

13. Herrmann, H.; Cerny-Reiterer, S.; Gleixner, K.V.; Blatt, K.; Herndlhofer, S.; Rabitsch, W.; Jäger, E.; Mitterbauer-Hohendanner, G.; Streubel, B.; Selzer, E.; et al. CD34(+)/CD38(-

) stem cells in chronic myeloid leukemia express Siglec-3 (CD33) and are responsive to the CD33-targeting drug gemtuzumab/ozogamicin. *Haematologica* **2012**, *97*, 219-226.
